# Gene functionalities and genome structure in *Bathycoccus prasinos *reflect cellular specializations at the base of the green lineage

**DOI:** 10.1186/gb-2012-13-8-r74

**Published:** 2012-08-24

**Authors:** Hervé Moreau, Bram Verhelst, Arnaud Couloux, Evelyne Derelle, Stephane Rombauts, Nigel Grimsley, Michiel Van Bel, Julie Poulain, Michaël Katinka, Martin F Hohmann-Marriott, Gwenael Piganeau, Pierre Rouzé, Corinne Da Silva, Patrick Wincker, Yves Van de Peer, Klaas Vandepoele

**Affiliations:** 1 CNRS, UMR 7232, Observatoire Océanologique, Banyuls-sur-Mer, France; 2 UPMC Univ Paris 06, UMR 7232, Observatoire Océanologique, Banyuls-sur-Mer, France; 3 Department of Plant Systems Biology, VIB, Technologiepark 927, B-9052 Ghent, Belgium; 4 Department of Plant Biotechnology and Bioinformatics, Ghent University, Technologiepark 927, B-9052 Ghent, Belgium; 5 Genoscope, CEA, Institut de Génomique, 2 rue Gaston Crémieux CP5706, 91057 Evry cedex, France; 6 Department of Biotechnology, Norwegian University of Science and Technology (NTNU), 7491 Trondheim, Norway †These two authors contributed equally to this work

## Abstract

**Background:**

*Bathycoccus prasinos *is an extremely small cosmopolitan marine green alga whose cells are covered with intricate spider's web patterned scales that develop within the Golgi cisternae before their transport to the cell surface. The objective of this work is to sequence and analyze its genome, and to present a comparative analysis with other known genomes of the green lineage.

**Research:**

Its small genome of 15 Mb consists of 19 chromosomes and lacks transposons. Although 70% of all *B. prasinos *genes share similarities with other Viridiplantae genes, up to 428 genes were probably acquired by horizontal gene transfer, mainly from other eukaryotes. Two chromosomes, one big and one small, are atypical, an unusual synapomorphic feature within the Mamiellales. Genes on these atypical outlier chromosomes show lower GC content and a significant fraction of putative horizontal gene transfer genes. Whereas the small outlier chromosome lacks colinearity with other Mamiellales and contains many unknown genes without homologs in other species, the big outlier shows a higher intron content, increased expression levels and a unique clustering pattern of housekeeping functionalities. Four gene families are highly expanded in *B. prasinos*, including sialyltransferases, sialidases, ankyrin repeats and zinc ion-binding genes, and we hypothesize that these genes are associated with the process of scale biogenesis.

**Conclusion:**

The minimal genomes of the Mamiellophyceae provide a baseline for evolutionary and functional analyses of metabolic processes in green plants.

## Background

Marine phytoplankton is responsible for about half of the photosynthetic activity on the planet [1], the second half being carried out by terrestrial plants. Two major traits differentiate these two classes of organisms. First, phytoplankton is essentially composed of unicellular organisms that have a high turnover; whereas terrestrial plants are renewed, on average, once every 9 years, the global phytoplankton population is replaced approximately every week [1]. Second, while photosynthesis is confined to specific organs of plants, often only a minor component of the plant biomass, in phytoplankton, photosynthesis essentially takes place in each cell. Phytoplankton populations are thus highly dynamic and may be able to adapt rapidly to changing environments. Even so, a global decline of photosynthetic micro-organisms over the past century has recently been reported [2], motivating research aimed at better understanding the global diversity of phytoplankton and how these species adapt to changing marine environment.

Phytoplankton is usually pragmatically classified according to size, from pico- (below 3 µm), nano- (3 to 8 µm) to micro-algae (above 5 to 8 µm), although these categories have no evolutionary significance. The eukaryotic fraction of picophytoplankton accounts for a modest part of the oceanic biomass, but nevertheless contributes an important part to primary production in many oceanic waters [3,4]. Among these picoeukaryotes, environmental diversity studies based on ribosomal gene sequences showed that small green algae, and notably the three genera *Bathycoccus*, *Micromonas *and *Ostreococcus*, are distributed worldwide and are numerically important in coastal areas. These three genera are characterized by their small size (1 to 2 µm), their rudimentary cellular organization (one mitochondrion and one chloroplast) and their small genomes (from 13 to 22 Mb). *Micromonas *[5] is a naked cell with one long flagellum whereas the two other genera are non-motile. *Ostreococcus *[6,7] is naked whereas *Bathycoccus *[8] is covered with scales. The complete genome sequences of two *Micromonas *[9], two *Ostreococcus *[10,11] and a low-light adapted strain of *Ostreococcus *(strain RCC809, available on the Joint Genome Institute web site) have been analyzed. The three genera belong to the order Mamiellales, in the class Mamiellophyceae [12,13], a monophyletic group in the phylum Chlorophyta. The ancestors of these micro-organisms emerged at the base of the green lineage and knowledge about them provides a baseline for exploring the evolution of this lineage, which also gave rise to terrestrial plants. Given their small cellular and genome sizes, they may reveal the 'bare limits' of life as a free-living photosynthetic eukaryotes, thus presenting a simple organization with very little non-coding sequences [14].

Here we report the analysis of the genome of one Mediterranean strain belonging to the genus *Bathycoccus *and its comparison with Mamiellales and other green algae, allowing a survey of the genome organization at the base of the green lineage. Although *Bathycoccus *was initially isolated from deep water (100 meters) [8], it has been frequently reported in various marine environments and seems an important component of the picoeukaryote compartment [15-18]. The availability of this genome, coupled to the development of new sequencing possibilities for metagenomes [19,20] from various marine environments, opens the way for comparative studies and to a better understanding of the adaptations of this organism to its environment(s).

## Results and discussion

### Characterization and phylogenetic position of the *Bathycoccus prasinos * RCC1105 strain

**Figure 1:**
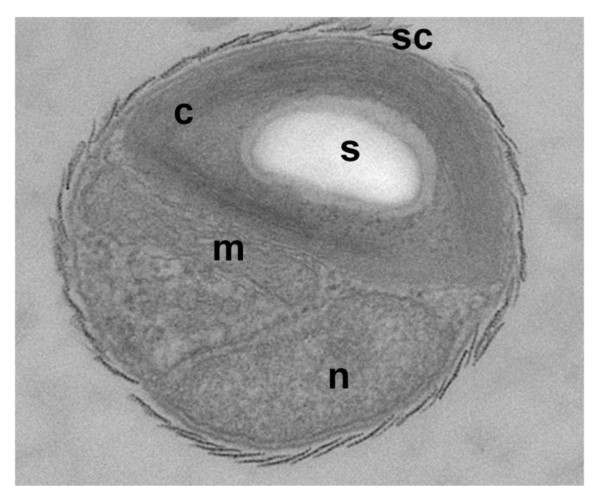
**Morphology of the *Bathycoccus prasinos *RCC1105 strain**. Morphological characterization of the *Bathycoccus *RCC1105 strain: EM picture of an exponentially growing *Bathycoccus *RCC1105 cell. Abbreviations: c, chloroplast; n, nucleus; s, starch granule; sc, scale covering the surface of the cell.

We isolated the *Bathycoccus prasinos *strain RCC1105 from a seawater sample from Banyuls' bay collected in January 2006. Contrary to the type strain described as *Bathycoccus prasinos *[8], which was isolated at a depth of 100 meters, RCC1105 was isolated from surface water (5 m). The strain RCC1105 has a typical *Bathycoccus *morphology with scales covering the cell (Figure 1) and we confirmed its taxonomic affiliation by PCR amplification of its 18S ribosomal gene. The complete genome of RCC1105 revealed two unlinked identical copies of the rDNA genes. Unlike the two previously reported *B. prasinos *isolates [8,21], these two ribosomal 18S genes were found to harbor an identical 433 bp long group I intron starting at position 551. Apart from this, the nucleotide sequence was strictly identical to the reference strain (GenBank: AY425315, FN562453). Self-splicing group I introns are widespread in nature, and have been recorded in the 18S rDNA of several other protists [22], including some within the green lineage, but, so far, not within the Mamiellales. All four *Bathycoccus *strains isolated from the Mediterranean bear this intron located exactly at the same splicing site. Phylogenetic analysis based on this small ribosomal subunit and on the internal transcribed spacer (ITS) confirmed that, in contrast to the two other Mamiellales' genera *Micromonas *and *Ostreococcus*, all *Bathycoccus *strains isolated to date comprise only one clade [12,13]. To confirm the phylogenetic position of *Bathycoccus *within the Mamiellales, we concatenated a set of 154 single-copy genes conserved in 13 species, including plants, and aligned them over 35,431 amino acids to construct a maximum likelihood phylogenetic species tree (Figure S1 in Additional file 1, and Additional files 2 and 3). The phylogeny obtained was well-supported and showed that the genus *Bathycoccus *is closer to *Ostreococcus *than to *Micromonas*.

### Global characteristics of the *Bathycoccus * genome

**Figure 2:**
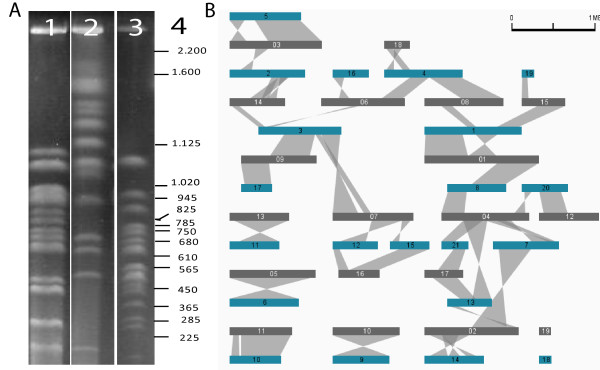
**Genome organization of the *Bathycoccus prasinos *RCC1105 strain**. **(a) **Pulse field electrophoresis of the genomes of *Bathycoccus prasinos *RCC1105 (lane 1), *Micromonas pussilla *(lane 2) and *Ostreococcus tauri *(lane 3) DNA fragment length based on the chromosomes of *Saccharomyces cerevisiae *(lane 4). **(b) **Synteny between *Ostreococcus lucimarinus *(blue) and *Bathycoccus prasinos *RCC1105 (grey) genomes.

The global characteristics of the *Bathycoccus *genome are similar to those observed in other Mamiellales except for its significantly lower GC content [17] (Table 1; Figures S2 and S3 in Additional file 1). The global genome size, measured by both pulsed field gel electrophoresis (Figure 2a) and sequencing, is intermediate (15 Mb) between *Ostreococcus *(12 to 13 Mb) and *Micromonas *(21 to 22 Mb), also reflecting an intermediate number of genes (Table 1; Table S1 in Additional file 1). Both sequencing and pulsed field gel electrophoresis also showed the genome to comprise 19 chromosomes, a number close to that found in other Mamiellales, and in other green algae despite the variation in genome size (Table 1; Table S1 in Additional file 1). The 15 Mb genome was sequenced at 22-fold coverage using a whole-genome shotgun sequencing approach, resulting in 126 contigs ranging from 3 to 1,353 kb. According to blast analysis, the 102 smallest of these contigs were bacterial contaminations, whereas the 24 remaining bigger contigs were part of the *Bathycoccus *genome (22 nuclear, 1 chloroplastic and 1 mitochondrial contig). Among the 22 nuclear contigs, six could be joined two by two, giving 19 scaffolds corresponding to 19 chromosomes observed by pulse field electrophoresis (Figure S3 in Additional file 1; Table S1 in Additional file 1; Additional file 4). Using intrinsic and extrinsic information, we predicted 7,847 genes in the nuclear genome (see Materials and methods), giving a high gene density similar to other Mamiellales. The validity of a majority of predicted genes was supported either by ESTs (approximately 46%) or by protein similarity (approximately 85%), and approximately 15% of them contain introns. Very few repeat sequences were found and no known or new transposable elements were detected (Table S1 in Additional file 1). The synteny observed between the chromosomes of *Ostreococcus *and *Bathycoccus *(Figure 2b; Figure S4 in Additional file 1) shows that the genome organization is globally better conserved between these two genera than with the genus *Micromonas*, in agreement with the phylogenetic analysis.

**Table 1:** Nuclear genome characteristics of green algae

Family	Species	Genome size (Mb)	G+C (%)	Chromosome number	Gene number
Prasinophyceae	*Bathycoccus *sp. RCC1105	151	48	19	7,847
Prasinophyceae	*Micromonas *sp. RCC299	20.9	64	17	10,286
Prasinophyceae	*Micromonas *sp. CCMP1545	21.9	65	19	10,587
Prasinophyceae	*Ostreococcus lucimarinus *clade A	13.2	60	21	7,805
Prasinophyceae	*Ostreococcus *sp. RCC809 clade B	13.3	60	20	7,492
Prasinophyceae	*Ostreococcus tauri *clade C	12.6	59	20	8,116
Trebouxiophyceae	*Chlorella *sp. NC64A	46	67	12	9,791
Chlorophyceae	*Chlamydomonas reinhardtii*	121	64	17	15,143
Chlorophyceae	*Volvox carterii*	138	56	14	14,520

Data from [9,10,24,68,69].

Based on the annotated gene sets of different land plants and green algae, sequence similarity searches were performed to group homologous genes into families (a family being defined as a set of two or more homologous genes; see Materials and methods). Subsequently, pan and core genome plots were built to quantify the number of shared and unique genes and families between different species (Figure S5 in Additional file 1). Comparing the set of core genes between different algal groups reveals that the smaller genome sizes of Mamiellales, as well as the lower number of genes, correspond both with the decrease of the average number of genes per family and with the number of families conserved within a specific clade. For example, whereas the number of gene families shared between all land plants, Chlamydomonales, and Trebouxiophyceae is 2,692, this number drops to 1,959 when including all Mamiellales species. Similarly, based on a set of core gene families conserved in both land plants and algae, the average gene family size is smaller for Mamiellales compared to Trebouxiophyceae or Chlamydomonales (average of 1.63, 1.78 and 1.93 genes per family, respectively). More than 500 gene families were found that were conserved between land plants and green algae but that were lost in all Mamiellales species (Figure S6 in Additional file 1). These families were enriched for functions related to zinc ion-binding and transport (ten families), UDP-glucosyltransferase activity (six families), vitamin ion binding (eight families) and sucrose and fatty acid metabolism (eight families) (Table S2 in Additional file 1). Although this pattern suggests a reduction of the functional gene repertoire, we also found more than 400 gene families that are specific to Mamiellales and found in all Mamiellales species. Whereas many of these Mamiellales-specific genes have unknown functions, three families related to drug transport and ten families including genes related to zinc ion binding were found (Table S2 in Additional file 1). Although rapid sequence evolution can interfere with the accurate detection of homologs using similarity searches, the observed pattern indicates a high turnover of zinc ion binding-related genes.

### Biological role and evolution of the big and small outlier chromosomes in *Bathycoccus * and in the Mamiellales

**Figure 3:**
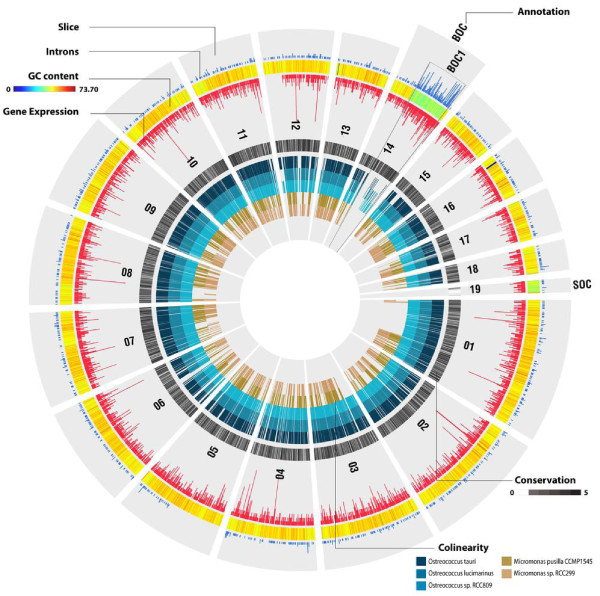
**Integrative and comparative view of the *Bathycoccus *genome showing both structural (GC content, introns, colinearity) and functional characteristics (gene expression, conservation)**. 'Slice' represents a single chromosome or region drawn using one gene per unit. 'Introns' and 'Gene Expression' denote the number of introns and uniquely mapped ESTs per gene, respectively. To improve legibility, an upper limit was set for the EST and intron count per gene by removing the top 2%, resulting in a threshold of 12 and 13 for intron count and gene expression, respectively. The GC content is plotted using a window size of 500 bp. The 'Annotation' track represents specific chromosomes or regions denoted by the different grey boxes; BOC and SOC refer to big and small outlier chromosome, respectively. 'Conservation' represents, for each gene, the number of Mamiellales species in which a BLAST hit can be found (E-value threshold 1e-05; range 0 to 5 species). 'Colinearity' shows for each gene if it resides in a genomic region showing colinearity with another Mamiellales species. The circle plot was drawn using the Circos circular visualization software [67].

Despite the low average GC content (48%; Table 1) of the *Bathycoccus *genome compared to other members of the Mamiellales (over 59%; Table 1), two outlier chromosomes were found, one 'big' (chromosome 14) and one 'small' (chromosome 19), with lower GC content (42%) compared to the rest of the genome (Table 2; Figure S7 in Additional file 1). This kind of organization was previously reported in *Micromonas *and *Ostreococcus *[9-11,23] and thus is a characteristic of all Mamiellales that have been sequenced so far. In all species, the atypical genomic features for the 'big' outlier chromosomes (BOCs) are restricted to a sub-region (referred to as BOC1) of the complete chromosome, whereas the whole length of the 'small' outlier chromosome (SOC) shows low GC content (Figure S7 in Additional file 1). However, although a BOC region was found for the 'low-light' *Ostreococcus *sp. RCC809 genome, which is available on the Joint Genome Institute website (unpublished), no clear SOC could be identified (Additional file 1). Whether this observation is biologically correct or the consequence of the applied sequencing approach, read filtering, or genome assembly remains currently unclear. Similar outlier chromosomes have not been found in other green algae such as *Chlamydomonas*, *Volvox *or *Chlorella*. In *Chlorella *low GC chromosome regions were reported [24], but these were, in contrast to those in the Mamiellales, scattered throughout different chromosomes. Outlier chromosomes are highly diverged in terms of gene content. Whereas most *Bathycoccus *chromosomes share, to some extent, a conserved genome organization with the other Mamiellales, both BOC1 (217 annotated genes) and SOC (72 annotated genes) lack colinearity (Figure 3), and this pattern is largely conserved between the outliers of the three genera. Many BOC1 genes share orthologs with other Mamiellales while SOC comprises mainly unknown, species-specific genes with few introns (26% of the SOC proteins have Gene Ontology functional annotation versus 71% for BOC1 genes and 44% for the rest of the genome; Figure 3; Figure S8 in Additional file 1). Additionally, phylogenetic estimations of the proportions of genes lacking plant orthologs yielded 75% (54/72) for SOC, 16% for BOC1 and 25% for normal chromosomal regions.

**Table 2:** Characteristics of the small outlier chromosomes for *Bathycoccus *and one *Micromonas *and one *Ostreococcus *species

Species	Chromosome number	Size (kb)	GC (%)	ORF number	Gene densities (bp/gene)	Identified genes	Sugar metabolism	Methylation enzymes	Other function
*Bathycoccus *sp.	19	146	42	72	2,031	34 (47%)	17 (24%)	7 (10%)	4 (6%)
*Ostreococcus lucimarinus*	18	149	53	78	1,915	32 (41%)	16 (21%)	5 (6%)	11 (14%)
*Micromonas *sp. RCC299	17	215	51	80	2,684	30 (38%)	14 (18%)	7 (9%)	9 (11%)

ORF, open reading frame.

### The big outlier chromosome in *Bathycoccus *

**Figure 4:**
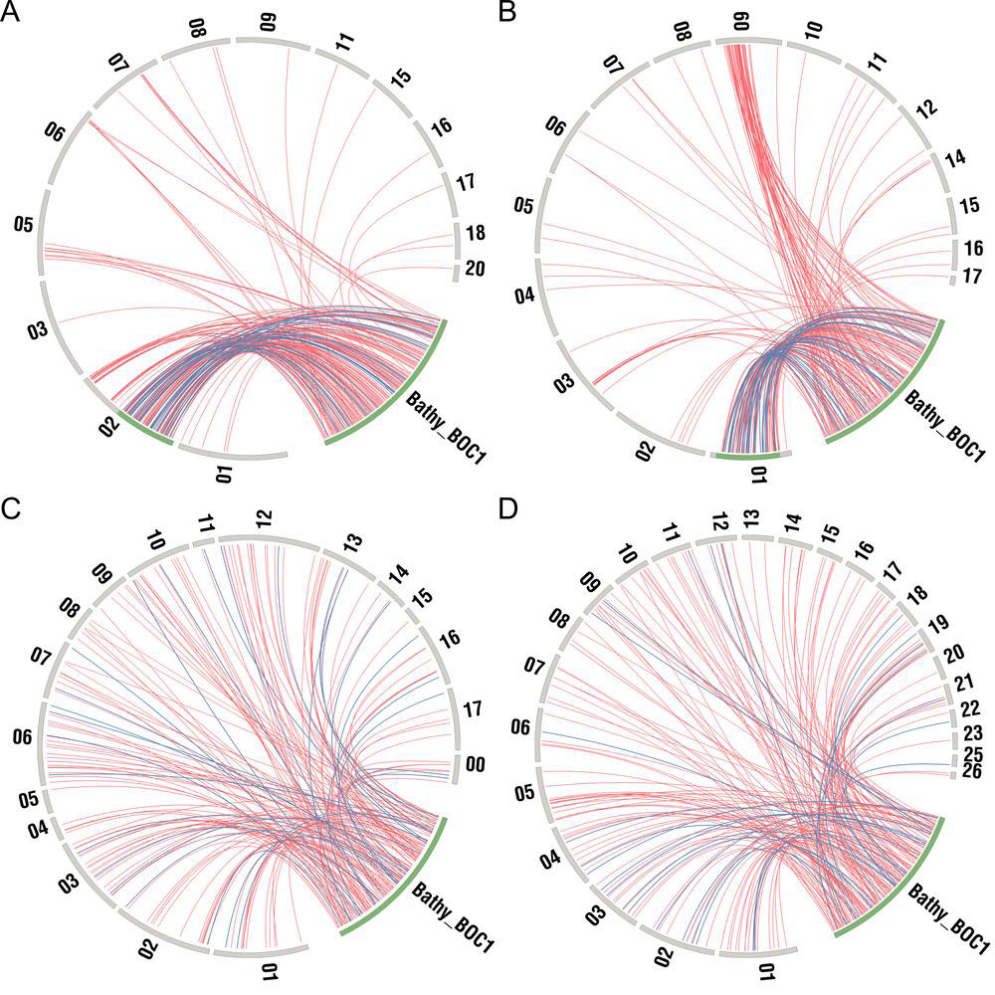
**Distribution of the *Bathycoccus *BOC1 orthologous genes in the genome of several other green alga species**. **(a-d) ***Bathycoccus *BOC1 orthologous genes in the genomes of *Ostreococcus tauri *(a), *Micromonas *sp. RCC299 (b), *Chlamydomonas reinhardtii *(c) and *Coccomyxa *sp. C-169 (d), each peripheral bar representing a chromosome. The *Bathycoccus *BOC1 region genes (lower right corner, labeled as 'Bathy_BOC1') are connected by red lines to their orthologs (curated as best BLAST hits) on the chromosomes of other species. Where a *Bathycoccus *gene also represents a BOC1 Mamiellales core gene (Table 3), the link is colored blue. Green bars show the BOC1 regions in *Bathycoccus*, *Micromonas *and *Ostreococcus*. The *Micromonas *sp. RCC299 region showing partial clustering of BOC1 orthologs lacks typical BOC1 features. This region has high GC content (66%), is not enriched for a high intron content and does not group highly expressed genes (Table S4 in Additional file 1). For the sake of legibility, all small scaffolds of *Chlamydomonas reinhardtii *that harbored a *Bathycoccus *BOC1 orthologous gene were joined together into a virtual chromosome 00 (scaffolds 19, 20, 22, 23, 24, 26 and 32).

The size of the *Bathycoccus *BOC is 663,424 bp. Fifty-two and seventy-eight percent of the *Bathycoccus *BOC1 genes having orthologs in other species were also located in the BOC in *Micromonas *and *Ostreococus*, respectively (Figure 4). In contrast, the locations of 29 BOC1 single-copy conserved gene markers (that is, genes having orthologs and located in BOC1 in all Mamiellales; Table S3 in Additional file 1) were scattered throughout the genomes in *Chlamydomonas*, *Volvox *and *Chlorella*, revealing that, despite the absence of colinearity, the clustering of the BOC1 genes is conserved and unique to the Mamiellales. These data suggest that BOC1 is a conserved genome property that was present in the last common ancestor of the Mamiellales. Genes located in the BOC1 region are over-represented in basic housekeeping functions like primary metabolism, gene expression, photosynthesis and protein transport (Figure S8 in Additional file 1; Table S3 in Additional file 1). To identify genomic features that are specific for the BOC1 region, the C-hunter tool (see Materials and methods) was applied to detect significant physical clustering of highly expressed genes and intron-containing genes on the different chromosomes (Table S4 in Additional file 1). C-hunter analysis revealed that the BOC1 region shows, in all species, a significant over-representation of EST-supported genes. Globally, 75% of all BOC1 *Bathycoccus *genes are EST supported versus 47% for non-BOC1 genes (Figure 3). After correcting for the overall 1.6-fold higher expression of BOC1 genes, BOC1 genes related to chromatin assembly, protein transport activity and signal transduction showed increased expression levels (Figure S8 in Additional file 1). To verify whether the high expression is a property of the low GC genomic BOC1 region (for example, due to a more open chromatin structure [25]), we checked the expression level of the genes on the other low GC chromosome, SOC. We found that SOC genes had no difference in expression level compared to the genes on the 17 other chromosomes. We further investigated whether this higher expression rate is an intrinsic property of the genes themselves, and estimated the expression levels for orthologs in *Chlamydomonas, Volvox *and *Coccomyxa *sp. C-169 (Figure S9A in Additional file 1). In all three species, BOC1 orthologs were also more highly expressed than other genes in the genome, suggesting that the higher expression of BOC1 genes in the Mamiellales is related to their function. Alternatively, this pattern might also be due to the global positive correlation, observed for all Mamiellales, between intron content and expression (Figure S9B in Additional file 1). Although the high expression of basic housekeeping BOC1 gene functions might yield increased metabolic rates and overall growth, it is not clear whether the physical clustering of BOC1 genes in the Mammiellophyceae lineage is based on adaptive gene relocation or constrained ancestral location [26].

The BOC1 region also displays structural specificities that are absent in the rest of the genome. Besides its low GC content, genes in the BOC1 region are split by many small (40 to 65 bp) AT-rich introns [10,11]. This feature is present in all of the sequenced Mamiellales genomes (Figure S10 in Additional file 1) and absent from the genomes of other green algae (Figure S10 in Additional file 1). There is no universal RNA-fold for these introns and no conserved sequence motifs (for example, branch points, splice sites) could be detected. Although the only intrinsic indication from their DNA sequences that they are introns comes from their AT-richness relative to the surrounding GC rich exons, their existence is clear from EST data. Consequently, the BOC1 region includes a high proportion of multiple exon genes, a feature absent in the rest of the genome (Table S2 in Additional file 1). In *Bathycoccus*, 103 of the 214 BOC1 genes harbor 330 introns, an intron content tenfold higher than in the rest of the genome (average of 1.54 and 0.15 introns per BOC1 and non-BOC1 gene, respectively).

In conclusion, the BOC1 region in the Mamiellales has unique structural characteristics: it represent one contiguous low(er) GC content region in the chromosome, flanked by two high(er) GC content regions at the extremities and carries between 193 and 633 genes depending on the species examined. The gene order within the region shows little colinearity between species and it encodes a high proportion of often vital housekeeping genes with elevated expression levels clustered together in a pattern unique to the Mamiellales (Figure 4). The biological reason for the existence of this region remains obscure, although its structural characteristics (shuffling of genes, small introns, low GC content) concur with the hypothesis that it may be a sex or species-barrier chromosome [27,28].

### The small outlier chromosome in *Bathycoccus *

The size of the *Bathycoccus *small outlier chromosome is 146,238 bp. compared to around 150 kb in *Ostreococcus lucimarinus *and 200 to 250 kb in *Micromonas*. The SOC average gene density in *B. prasinos *is slightly lower than that observed in the other chromosomes (72 genes with an average of 2.0 kb per gene in SOC compared to 1.7 kb per gene in the global genome), with a similar expression level based on EST counts. Only 44% of the genes in SOC have a potentially identified function compared to 77% in other chromosomes. Furthermore, up to 75% of the SOC genes have no known plant orthologs, in sharp contrast to most other chromosomes, where most genes share green lineage descent. Last but not least, in *Bathycoccus*, 24 of the 34 SOC genes having an identified function group in two categories. The first group encodes enzymes involved in metabolism of glycoconjugates (17 genes), mainly glycosyltransferase (12 genes), and the second is related to methyl transferases (7 genes). These features are globally similar in the other known Mamiellales SOCs, where the same two dominant gene functions were found (Table 2). However, despite their common function, no synteny and almost no orthologous relationships could be established between the SOCs of the different Mamiellales' species, suggesting a more functional convergence than a common phylogenetic origin. To explain the presence of such genes in SOCs, an alien origin of these chromosomes was proposed, which could have yielded some selective advantages in cell surface processes, potentially related, for example, to defense against pathogens or other environmental interactions [10]. However, since SOCs and BOCs have now been found in all sequenced mamiellophycean genomes, it is likely that their lower GC composition, higher proportion of specific genes and higher evolution rates [9,29] are being maintained by the same evolutionary pressure in all of these species. Interestingly, a paper on the cyanobacteria *Prochlorococcus *describes how variable genomic islands showing similar characteristics to those found in SOCs (low number of orthologs, a high level of horizontal gene transfer (HGT) and a high fraction of sugar-modifying enzymes, methyl transferases and membrane associated proteins) are involved in resistance to viruses [30]. The viral resistance determined by these genomic islands induced a fitness cost measured either by a reduced growth rate and/or a more rapid infection by other viruses. The three genera *Bathycoccus*, *Micromonas *and *Ostreococcus *are the microalgae tested, which are among the most attacked by viruses [31], and viral resistance phenomena showing similar characteristics to what is reported for *Prochlorococcus *(reduced growth rate and higher infection rate by other viruses) have been reported to occur frequently [32]. It is tempting to link this unusual high viral sensitivity and the ability to develop rapid and frequent resistance to these attacks to the presence of SOCs. Interestingly, two other Mamiellales species (*Mamiella *sp. or *Mantoniella squamata*) were tested recently and did not show this high viral sensitivity (N Simon, personal communication). It can be predicted that if our hypothesis on the link between SOC and viral hypersensitivity/resistance is correct, these species should not present a SOC-like structure in their genome.

### Phylogenomics suggests many horizontal gene transfers

**Figure 5:**
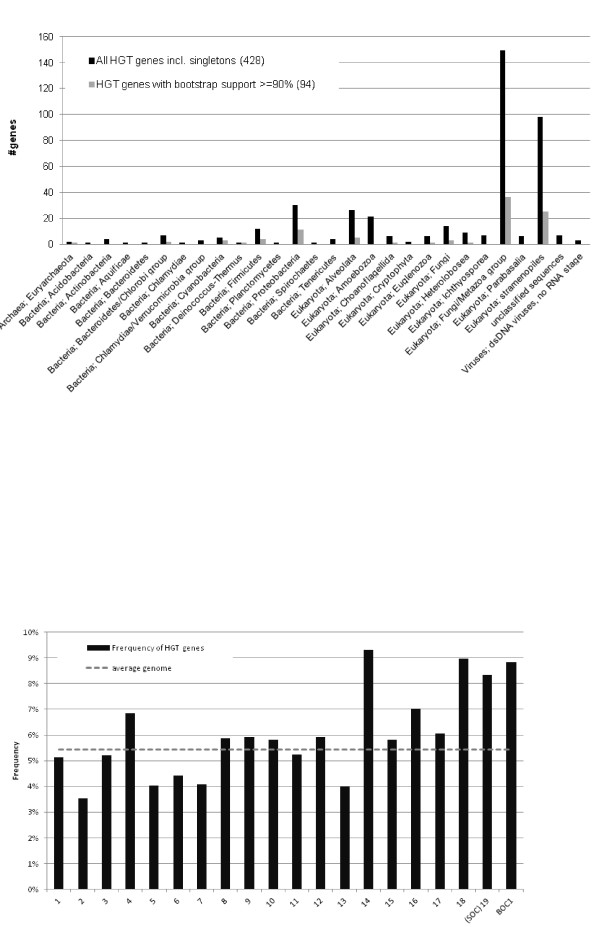
**Potential horizontal gene transfer in *Bathycoccus***. **(a) **Taxonomic distribution of horizontal gene transfer (HGT) genes identified using BLAST and by phylogenetic analysis of each gene (excluding genes with a multi-kingdom punctuate distribution). Only taxonomic groups including multiple genes are displayed (for a complete overview, see Table S5 in Additional file 1). **(b) **Frequency of 428 HGT genes over the different chromosomes. The last bin reports the fraction of HGT genes in the BOC1 region (a subset of chromosome 14).

Based on the observation that no plant homologs could be found for many annotated *Bathycoccus *genes, a systematic analysis was performed to unravel their origin. Since plain sequence similarity search strategies are insufficient to reliably trace a gene's evolutionary history [33,34], a two-step comparative approach was applied to identify putative HGT events. After comparing each *Bathycoccus *protein sequence against the National Center for Biotechnology Information (NCBI) protein database, 6,550 phylogenetic trees were constructed and conflicts between the gene and organism phylogeny were determined. Whereas clustering patterns where the nearest neighbor in the tree corresponds with a homolog from a species outside the plant lineage were scored as HGT, in some cases ancestral gene duplication followed by differential gene loss or artifacts of phylogenetic reconstruction methods due to unusual modes of protein evolution could yield misleading results [35]. There were 428 genes (6%) that clustered with a homologous gene from a species outside the green lineage, whereas the remaining genes grouped with Viridiplantae genes (70%) or did not show any significant similarity. Among the 428 putative non-Viridiplantae genes, 80% were of non-green eukaryotic origin while 17% were bacterial orthologs (Figure 5a). For the 354 non-green eukaryotic genes, a high proportion came from Metazoa and Stramenopiles (42% and 28%, respectively). Gene Ontology enrichment analysis showed that around 50% of the non-Viridiplantae genes (including prokaryotic genes) were involved in metabolism. Focusing on the most enriched categories revealed genes involved in zinc ion binding (61, 6-fold enrichment), sialyltransferase activity (27, 12-fold enrichment), glycosylation (27, 11-fold enrichment) and ankyrin repeats (5-fold enrichment) (these observations are discussed further in the following section). Application of conservative selection criteria (retaining only phylogenetic trees with bootstrap support >90% and more than 50% protein alignment coverage) yielded 79 genes with non-plant nearest neighbors (Table S5 in Additional file 1), which we propose might originate from HGTs, either from eukaryotes (82%) or prokaryotes (18%). Most of these 98 highly probable HGTs (43%) show unknown functions and the others, both originating from pro- or eukaryotes, show metabolite functions (based on similarities with protein domains). The absence of detectable eukaryotic HGT in *Arabidopsis thaliana*, our negative control, suggests that this finding is not an artifact of the method. Using the same approach, previous putative large-scale HGTs have been reported in the available nuclear diatom genomes [36,37], both from bacteria (784 genes in *Phaeodactylum tricornutum*) or from the green lineage (>1,700 genes). However, although no other 'eukaryotic' potential HGTs are discussed in these papers, orthologous genes shared with other eukaryotic lineages were also described. The presence of green genes in diatoms has been explained by endosymbiotic gene transfers and an alternative hypothesis would be that the presence of stramenopile genes in *Bathycoccus *may reflect an opposite gene flow from diatom-like cells to Mamiellales. This hypothesis seems unlikely, however, because most of the stramenopile genes found in *Bathycoccus *are specific to this species and are not found in other Mamilelalles genomes. Alternatively, this mosaic gene repertoire could be the consequence of (i) parallel or convergent molecular evolution or (ii) the evolution through gene loss of a large ancestral genome, with massive and selective gene losses in all Mamiellales descendants, concurrent with genome reduction. However, this scenario is less parsimonious compared to HGT and, again, seems unlikely because of the phylogenetic breadth of the selectively retained genes (bacterial and from different supergroups of the eukaryotic tree of life).

In line with a recent report about the acquisition of ice-binding proteins in sea ice diatoms from prokaryotic origin [38], it is tempting to speculate that the HGT genes contribute new functional properties to the *Bathycoccus *genome. The analysis of a large DNA virus in *Ostreococcus tauri *suggested that the capture of host DNA in viral genomes could represent a mechanism for the transfer of genes between eukaryotic cells [39]. This idea was confirmed by the additional sequencing of four double-stranded DNA marine prasinovirus genomes (infecting *Bathycoccus*, *Micromonas*, and *Ostreococcus*), showing that these viruses encode a gene repertoire of certain amino acid biosynthesis pathways never previously observed in viruses that are likely to have been acquired from lateral gene transfer from their host or from bacteria [40]. A similar eukaryotic phytoplankton-virus system was also described in *Emiliania huxleyi*, mediating the transfer of seven genes related to sphingolipid biosynthesis [41].

To verify whether specific genomic regions or chromosomes would be more likely to harbor genes arriving via HGT, we estimated the number of HGT genes per chromosome. We observed that transferred genes were more or less equally distributed over the different chromosomes, except for the low GC outlier chromosomes, which contained higher fractions of HGT genes (BOC1 and SOC contain 1.63 and 1.54 times more HGT genes compared to the genome-wide average; Figure 5b). Different possibilities for the increased abundance of HGT on the outliers include, for example: (1) they may have specific sequence features that can serve to integrate HGT genes that are subsequently re-arranged and embedded in other locations in the genome; (2) it may reflect a lower density of essential gene functionalities in outliers, which could thus support a higher density of random insertions; or (3) there might be a lower level of recombination on these chromosomes, reducing the rate of removal of deleterious alleles via sexual recombination. None of these scenarios are mutually exclusive.

In the *Bathycoccus *genome, the gene copy number is highly expanded for four specific gene families, phenomena not found (or at very low copy number expansion) in other Mamiellales or other algae (Table 3). Of these, two are involved in the metabolism of sialic acids, that is, sialyltransferases (69 gene copies) and sialidases (23 gene copies), the two others being ankyrin-repeat proteins (149 gene copies) and zinc finger proteins (48 gene copies) (Table 3). Among these 289 gene copies, 105 (36%) are represented within the 428 probable genes acquired by HGT, representing 24% of them.

**Table 3:** Expanded gene families in the *Bathycoccus *genome

Gene family^a^	Copy number in *Bathycoccus prasinos*	Copy number in *Micromonas *sp. CCMP1545	Copy number in *Micromonas *sp. RCC299	Copy number in *Ostreococcus lucimarinus*	Copy number in *Ostreococcus *sp. RCC809	Copy number in *Ostreococcus tauri*
Glycosyl transferase, family 29 (IPR001675)	78	0	1	0	2	0
HOM000519	43	0	0	0	0	0
HOM002813	10	0	0	0	0	0
HOM005062	10	0	0	0	0	0
HOM007941	6	0	0	0	0	0
Ankyrin repeats (IPR020683)	186	124	107	74	55	67
HOM000035	149	56	9	17	6	6
Sialidase/neuraminidase (IPR011040)	23	1	0	0	0	0
HOM002557	17	0	0	0	0	0
HOM005056	5	0	0	0	0	0
Zinc finger, C2H2	53	29	35	19	3	17
HOM000293	48	5	4	1	1	1

^a^Protein domain description including InterPro identifier. HOM identifiers refer to gene families in pico-PLAZA [62].

### Sialic acid metabolism in *Bathycoccus *

**Figure 6:**
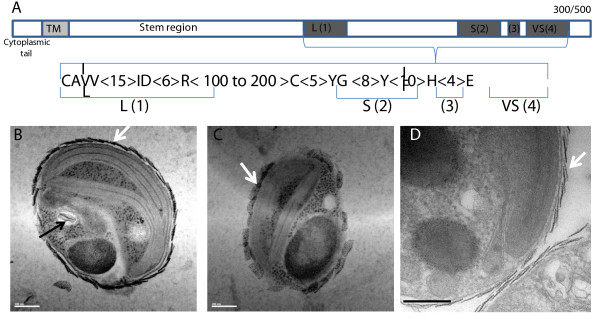
**Sialyltransferase gene family and external scales covering *Bathycoccus***. **(a) **Structural organization of the *Bathycoccus *RCC1105 sialyltransferase gene family. TM, transmembrane domain. L(1), S(2), (3) and VS(4) correspond to the four metazoan consensus motifs described for this gene family [43,44]. Letters in the scheme below are the amino acid one-letter code with alternative possibilities for positions indicated between brackets. **(b-d) **Details of external scales covering *Bathycoccus *RCC1105 cells; white arrows indicate positions of external scales around the plasmic membrane while the black arrow indicates one intracellular scale inside a vesicle.

The two enzyme families involved in the metabolism of sialic acids are not present in other known green algae genomes, and both gene families are dispersed all along the *Bathycoccus *genome without evident clustering or tandem duplication. Although, on average, 15% of the genes have introns in *Bathycoccus*, no introns (except three genes; Figure S11 in Additional file 1) were found in any gene from both families. Genes annotated as sialyltransferases correspond to glycosyltransferases family 29 in the CAZy classification, which comprises enzymes able to transfer sialic residues during glycosylation of proteins or lipids [42]. All the *Bathycoccus *sialyltransferases showed a metazoan taxonic affiliation and none of them gave significant hits with bacteria. These enzymes are type II single pass membrane proteins usually known to be anchored in the Golgi membranes [43,44]. A potential hydrophobic transmembrane domain was detected on the amino-terminal extremities of all the *Bathycoccus *sialyltransferases (Figure 6a). For almost all the 69 genes (only 19 are known in human), the sialyltransferase domain is located in the carboxy-terminal part of the protein, whereas the amino-terminal domain is composed of a highly variable stem region (Figure 6a). Although the existence of complete and active sialyltransferases in plants is still a matter of debate [45], all four metazoan consensus motifs were found in the *Bathycoccus *genes.

The second gene family includes sialidases (or neuraminidases), which are enzymes cleaving the terminal sialic acid residues from glycoproteins or glycolipids. Again, this gene expansion is specific to *Bathycoccus*. In contrast to the previous family, no clear domain organization could be defined in sialidases, but some key amino acids known to be involved in the catalytic activity are conserved in the *Bathycoccus *family. The taxonomic origin of the sialidases is less clear than that for the sialyltransferases discussed above, and could correspond to either metazoans or bacteria. For sialidases, scores are globally weak and best blast hits are found mostly with hypothetical proteins either from the choanoflagellate *Monosiga brevicolis *or from the green alga *Chlorella variabilis *(where only one sialidase has been annotated).

The expansion of these two enzyme families prompted us to look for specific potential 'sialic acid' metabolism in *Bathycoccus*. The composition of flagellar scales in *Scherffelia dubia *(phylum Chlorophyta, class Chlorodendrophyceae) was found to be a mix of acidic polysaccharides having similar structures to sialic acids [46]. Although the chemical nature of the scales covering the *Bathycoccus *cell membrane is unknown, it is tempting to establish a correlation between the potential biosynthetic pathway of these scales and the expansion of gene families involved in the metabolism of sialic acids. Furthermore, we confirmed previous electron microscopy studies [8,47] showing that, in *Bathycoccus *as in other Mamiellophyceae, scale biosynthesis occurs inside intracellular vesicles with striking resemblance to Golgi vesicles (Figure 6b-d); that is, in agreement with the notion that they might be produced by sialyltransferases located at the luminal side of intracellular vesicles. Scales almost identical to those of *B. prasinos *are observed in the more closely related *Mantoniella squamata *[48], where they are also extruded to the surface after transport via the Golgi body [49-51].

### Other *Bathycoccus * expanded gene families

One of the two other highly expanded gene families in the *Bathycoccus *genome are ankyrin-repeat proteins (149 gene copies). This family is also expanded, although to a lesser extent, in the *Micromonas *strain CCMP1545 (56 copies), whereas only very few copies were detected in other Mamiellales (Table 3). These genes have ankyrin repeats located in the carboxy-terminal part of the protein whereas the amino-terminal part has no hit in GenBank. There are also many other ankyrin repeats containing genes in *Bathycoccus *as in both plants and microalgae, but associated with different protein domains that often have predicted functionalities. Indeed, the ankyrin repeat is considered as one of the most common protein-protein interaction motifs in nature [52]. The 149 *Bathycoccus*-specific genes were not distributed randomly among chromosomes, with the bigger chromosomes having few copies, whereas chromosomes 12 or 19 bear many tandem duplicated genes (Figure S12 in Additional file 1). No obvious function can be attributed to these genes. However, by analogy with the human membrane-associated ankyrin, which is responsible for the attachment of the cytoskeleton to the plasma membrane, it is possible that a number of these genes might function in some way to bind extracellular scales to the plasmic membrane, although experimental evidence is lacking. It has been shown, however, by electron microscopy coupled to immunogold that scales in *Scherffelia dubia *are linked to the membrane by glycoproteins [46]. In addition, in *Tetraselmis striata *(Chlorodendrophyceae) some scale-associated glycoproteins may provide connections between scales and the underlying flagellar membrane [50].

The last group of expanded genes in *Bathycoccus *are zinc finger proteins. There are many zinc finger proteins in microalgae and in plants, but the family specifically expanded in *Bathycoccus *is most related to the C_2_H_2_-type zinc finger DNA-binding domain of certain integrases, which share a common alpha/beta two-layer sandwich core structure. The typical organization of the 48 copies identified in the *Bathycoccus *genome (Table 3) includes a short amino-terminal part (around 20 to 40 amino acids) followed by a strongly acidic region (10 to 20 amino acids) and by 2 to 6 C_2_H_2 _domains. Zinc finger proteins were originally identified as DNA-binding domains, although a growing body of evidence suggests an important and widespread role for these domains in protein binding. There are even examples of zinc fingers that support both DNA and protein interactions, and, globally, C_2_H_2 _protein-protein interactions are proving to be more abundant than previously appreciated [53].

The most parsimonious explanation for the abundance of the four expanded gene families would be an initial single HGT event followed by expansion in the *Bathycoccus *genome. The potential function of these four gene families and their expansion only in *Bathycoccus *also suggest that they could all be involved in the biosynthesis, exportation and fixation of the scales around the external membrane, and possibly for protection of the cell. Several other members of the Mamiellales have morphologically similar scales around the cells, but they are absent in the two genera *Micromonas *and *Ostreococcus*. The most parsimonious evolutionary scenario to explain these observations is that the scale synthesis pathway was acquired by the ancestor of the Mamiellales (or even before) and has been lost in the two naked genera. This scenario predicts that similar gene family expansions should be found in the genomes of other scaled Mamiellaophyceae but not in *Micromonas *and *Ostreococcus*. This is the case for *Micromonas *and *Ostreococcus*, but the genome sequences of other scaled species are not yet available.

## Conclusions

Mamiellophyceae, and more particularly the three genera *Bathycoccus*, *Micromonas *and *Ostreococcus*, are dominant in different marine areas, where they can play an important role in the primary biomass production. However, the ecological importance of *Bathycoccus *has probably been overlooked these past years, although it was sporadically mentioned in several studies [5,16-18]. The availability of this genome, coupled to the development of new sequencing possibilities for metagenomes [19,20] from various marine environments, opens the door to future comparative studies and to a better understanding of the adaptations of the organisms to their environment.

## Materials and methods

### *B. prasinos * RCC1105 genome and EST sequencing and annotation

The sequenced strain *B. prasinos *RCC1105 was isolated in the bay of Banyuls sur mer at the SOLA station (Additional file 1). The genomic DNA was extracted from cell pellets containing a collective total of 6.4 × 10^10 ^cells, using a cetyl trimethyl ammonium bromide protocol (adapted from [54]). The *Bathycoccus *genome was sequenced using Sanger technology on three independent shot-gun libraries with insert sizes of 3 (TK0AAA, vector pcdna2.1 (BstXI)), 10 (TK0AAB, vector pCNS (BstXI)) and 50 kb (TK0ACA, vector pBeloBAC11 (HindIII) and TK0ACB, vector pBeloBAC11 (BamHI)), resulting in 230,496 reads (180 Mb), 118,070 reads (152 Mb) and 10,368 reads (14 Mb), respectively. After trimming, read numbers were 223,577 reads (174 Mb) for the 3 kb library, 112,842 reads (145 Mb) for the 10 kb library and 8,189 reads (11 Mb) for the 50 kb library, and represented a coverage of 22-fold from 330 Mb of sequenced DNA. The data were assembled using the Genoscope pipe-line that includes the software Arachne 3.0 [55]. ESTs were sequenced from a *Bathycoccus *culture grown to log phase (10^7 ^cells/ml), harvested by centrifugation and the cell pellets were immediately flash frozen in liquid nitrogen. The total RNA was extracted using the TriReagent (Sigma-Aldrich, Saint-Quentin, France) protocol and mRNAs purified using Poly(A)Purist (Ambion-Applied Bioystems, Saint Aubin, France). Complementary DNAs were constructed and cloned using the CloneMiner procedure (InvitroGen, Saint Aubin, France) with some minor modifications. EST sequences were obtained using pyrosequencing technology developed by Roche (Boulogne-Billancourt, France). A total of 253,791 EST reads were processed through the Genoscope EST pipeline. Short (<60 bp) and low complexity sequences were identified and removed. Clustering and assembly of all 251,875 filtered EST reads resulted in 8,370 EST consensus sequences.

The genome was annotated using the EuGene [56,57] gene finding system with Splice- Machine [58] signal sensor components trained specifically on *Bathycoccus *datasets. The functional annotation resulted from the synthesis of InterPro and the BLASTP hits against the non-redundant UniProt database. Gene Ontology assignments were derived from the InterPro results. Gene Ontology enrichment analysis was performed using the hypergeometric distribution with Bonferonni correction for multiple hypothesis testing and corrected *P*-values <0.05 were retained as significant. The resulting database is publicly available at [59] in a format that includes browse and query options and the genome has been submitted to GenBank.

### Comparative sequence and expression analysis

Starting from all protein-coding genes from the included species (Table 1), only retaining the longest transcript if alternative splicing variants exist, protein sequences were used to construct gene families by applying sequence-based protein clustering. First, an all against all sequence comparison was performed using BLASTP, applying an E-value threshold of 1e-05 and retaining the best 500 hits [60]. Next, the complete sequence similarity graph was processed using Tribe-MCL (mclblastline, default parameters except I = 2 and scheme = 4) to identify gene families. A set of 154 single-copy core gene families was used to construct the phylogenetic tree depicted in Figure S1 in Additional file 1 (see also Additional files 2 and 3).

The boundaries of all Mamiellales BOC1 regions were manually delineated based on gene coordinates, gene family information and GC content (Table S2 in Additional file 1). For non-Mamiellales, a 'virtual' BOC1 region was created by taking the best BLASTP hit for each *B. prasinos *RCC1105 BOC1 gene. Putative BOC1 Mamiellales core gene families (Figure 6, blue lines) were identified by first retaining only those families that contain at least one protein for each Mamiellales species. Next, each family was aligned and manually curated. This was done by inspecting and correcting, if necessary, the structural and functional annotation (NCBI BLAST results plus InterProScan) of all cluster members. For *Ostreococcus *sp. RCC809 no SOC could be identified in the current draft genome assembly (Additional file 1).

### Comparative genomics

To detect co-linearity within and between species, i-ADHoRe 3.0 was used (Additional file 1) [61] and all chromosomes from all species were compared against each other and significant colinear regions were identified. All gene colinearity can be browsed using the pico-PLAZA comparative genomics platform [62]. i-ADHoRe was run with the following settings: alignment_method gg, gap_size 30, cluster_gap 35, q_value 0.9, prob_cutoff 0.0001, anchor_points 5 and level_2_only false.

EST databases were retrieved from their respective public repositories and mapped on the Mamiellales genomes using GenomeThreader [63] with a minimum alignment score threshold of 0.95 and minimum transcript coverage of 0.89. Only uniquely mapped ESTs were retained and assigned to genes. When an EST with no strand information overlapped with two adjacent genes, it was assigned to the gene with the highest overlap. For the BOC expression analysis global gene, EST counts were first summarized per functional category. In a second stage, expression enrichment was determined by comparing for each functional category the fraction of BOC expressed genes against the overall fraction of BOC expressed genes (denoted 'relative BOC expression enrichment' in Figure S8 in Additional file 1).

### Analysis of potential horizontal gene transfer

For each protein-coding gene a BLAST sequence similarity search was performed against the NCBI protein database, which contains the proteins of all sequenced *Ostreococcus*, *Micromonas *and *Chlamydomonas *species (E-value <1e-05). Starting from a selection of BLAST hits a phylogenetic approach was used to identify the putative origin of all genes. Briefly, good hits (20% top hits relative to the best Bit score excluding query self-hits) were retained per gene, protein sequences and detailed taxonomic information was retrieved, a multiple sequence alignment was generated using MUSCLE and a maximum likelihood phylogenetic tree was constructed using PhyML (100 bootstrap sets, WAG model, kappa estimated, 4 substitution rate categories, gamma distribution parameter estimated, BIONJ starting tree, no topology, branch lengths and rate parameter optimization). For each query gene the corresponding tree topology was investigated to identify the nearest neighbor gene/clade, including bootstrap support, and determine the nearest neighbor taxonomic information. Genes showing complex punctuate patterns [64] (that is, clustering with homologs from different phyla outside the Viridiplantae; labeled 'multi-kingdom' in Table S5 in Additional file 1) were excluded. Singletons refer to genes for which no phylogenetic analysis could be done because they only have a single BLAST hit based on the 20% top hits. Nearest neighbors with bootstrap support >90% and gene coverage of 50% or more in the multiple alignment were scored as reliable HGT genes to estimate the fraction of eukaryotic origin. Although the low number of HGT genes found in *Arabidopsis *does not serve as a perfect negative control for the detection of HGT in unicellular green algae, it suggests that, when applied to a full set of proteins of a specific organism, this approach gives a conservative estimate of putative transfer events with a low number of false positives. To verify if, for some HGT genes, homologous genes exist in other algae that were missed during the process of gene annotation, a systematic sequence similarity search (using tblastn, E-value threshold 1e^-05 ^against intergenic sequences of *O. tauri*, *O. lucimarinus*, *Ostreococcus *RCC809, *M. pusilla *and *C. reinhardtii*) revealed that, on average, no homologous locus could be found for 93% of the HGT genes. A list of all HGT genes together with protein alignment and phylogenetic tree statistics is available in Additional file 5.

### C-hunter analysis

Four functional categories (two types with two subdivisions each) were defined and genes were assigned to each class, if applicable. The first type of functional category describes the expression state of a gene (based on uniquely mapped ESTs; is a gene expressed (number of ESTs >0) or highly expressed (number of ESTs >2)) while the second type describes the intron content of a gene (contains an intron (number of introns >0) or contains a 'lot' of introns (number of introns >2)). C-hunter [65] software was used to identify, in all genomes, significant clusters of genes belonging to one of the four functional categories. The C-hunter thresholds for each category subdivision were determined by reviewing the average expression and intron content of all Mamiellales genes. C-hunter was run with the following parameters: <C-hunter categories.go genome.index genome.go 2 80 80 0.001 50 T chunter output>.

### Accession numbers

Sequence data from this article (the genome of *B. prasinos *RCC1105) can be found in the EMBL/GenBank data libraries under accession number [FO082258] (mitochondrion), [FO082259] (chloroplast), [FO082278] (chromosome 1), [FO082277] (chromosome 2), [FO082276] (chromosome 3), [FO082275] (chromosome 4), [FO082274] (chromosome 5), [FO082273] (chromosome 6), [FO082272] (chromosome 7), [FO082271] (chromosome 8), [FO082270] (chromosome 9), [FO082269] (chromosome 10), [FO082268] (chromosome 11), [FO082267] (chromosome 12), [FO082266] (chromosome 13), [FO082265] (chromosome 14), [FO082264] (chromosome 15), [FO082263] (chromosome 16), [FO082262] (chromosome 17), [FO082261] (chromosome 18), [FO082260] (chromosome19). The annotation of the genome can be found at the BOGAS web site [66]. EST data are available at the ENA database (accession number ERA148021) and raw genome sequencing data are available at the Trace archive database of the NCBI under the query: species_code="BATHYCOCCUS SP. BAN7".

## Abbreviations

BOC: big outlier chromosome; bp: base pair; CCMP: Center for Culture of Marine Phytoplankton; EST: expressed sequence tag; HGT: horizontal gene transfer; NCBI: National Center for Biotechnology Information; RCC: Roscoff Culture Collection; SOC: small outlier chromosome.

## Competing interests

The authors declare that they have no competing interests.

## Authors' contributions

All authors have read and approved the manuscript for publication. ED, H-MMF, JP, MK, CDS, and AC performed research and sequencing. HM, BV, SR, MVB, GP, PR, and KV analyzed the data and wrote the paper. NG, PW, and YVdP wrote the paper. HM and KV designed the research.

## Supplementary Material

Additional file 1 **Supplementary materials and methods, figures and tables**. Click here for file

Additional file 2 **The 154 single-copy core gene families in the green plant lineage**. Click here for file

Additional file 3 **Alignment of 154 single-copy core gene families in the green plant lineage**. Click here for file

Additional file 4 **Statistics of the genome shotgun sequencing**. Click here for file

Additional file 5 **Details of maximum likelihood phylogenetic trees describing *B. prasinos *RCC1105 HGT genes**. Click here for file
