# Supplementary material for: Gene functionalities and genome structure in Bathycoccus prasinos reflect cellular specializations at the base of the green lineage
Source: Genome Biol. 2012 Aug 24;13(8):R74. doi: 10.1186/gb-2012-13-8-r74 (PMC3491373; doi:10.1186/gb-2012-13-8-r74)
Supplement: Additional file 1 — Supplementary materials and methods, figures and tables. [file gb-2012-13-8-r74-S1.DOCX]

**Supplementary Methods**

***Bathycoccus prasinos* RCC1105 genomic DNA.** The sequenced strain *Bathycoccus prasinos* RCC1105 was isolated in the bay of Banyuls sur mer at the SOLA station (42°29'3N; 3°8’7E) at 3 metres depth on January 2006 and purified by plating out to ensure its clonality. The strain was treated with an antibiotic cocktail until no contaminating bacteria could be detected by flow cytometry during the time of the culture. The cells were grown in the Keller medium [1] and were harvested during the exponential growth phase at a concentration of 4.10^7^ cells/ml (see Fig. S2) by centrifugation for 20 min, 8,000 g, 4°C, flash frozen with liquid nitrogen, and stored at -80°C. The genomic DNA (both nuclear and organellar) was extracted from cell pellets containing a total of 6.4x10^10^ cells, using a CTAB protocol (adapted from [2]). The quality of the purified genomic *Bathycoccus* DNA was monitored with a wavelength absorbance scan and electrophoresis on a 1% 1X TBE agarose gel compared to varying amounts of lambda phage DNA.

**ESTs sequencing.** ESTs were sequenced from a *Bathycoccus* culture grown to log phase (4.10^7^ cells/ml, see Fig. S1), harvested by centrifugation and the cell pellets were immediately flash frozen in liquid nitrogen. The total RNA was extracted, polyA RNAs (mRNAs) were purified and non normalized cDNA libraries were prepared. EST sequences were obtained using pyrosequencing technology developed by Roche and a total of 253791 GSflx EST reads were processed. The gene expression level was extrapolated from the number of reads obtained for each mRNA. This method is an indirect proxy for the quantification of gene expression which can be used only from non-normalized cDNA libraries. This semi-quantitative method has been used for the approximation of the gene expression in the *Chlorella* genomes [3].

**Genome annotation and detection transposable elements detection.** The data sources used to complement the *ab initio* part of EuGene were composed of *B. prasinos* RCC1105 expressed sequence tags (ESTs), protein databases (TAIR10, *O. lucimarinus* proteome and SwissProt), and the other Mamiellales raw genomic sequences [4] (using the RepBase library [5]), LTRharvest [6] +LTRdigest [7], LTR_seq (http://eecs.wsu.edu/~ananth/sofware.htm), a BLASTP against all TE-related NRPROT proteins (E-value threshold 1e^-05^) and a detailed HMMer scan using all profiles from the Gypsy Database [8]. Repeats were detected using RepeatMasker (low-complexity regions and simple repeats) and findpat [9] (exact repeats>40nt). Noncoding genes were detected using an ensemble approach of RepeatMasker [10], RNAmmer [11], tRNAscan-SE [12], INFERNAL [13] and BLASTN (using *O. tauri* RNA data).

**Phylogenetic position *Bathycoccus prasinos* RCC1105.** Based on phylogenetic profiles present in the pico-PLAZA database (http://bioinformatics.psb.ugent.be/pico-plaza/), which represent the number of gene copies per family and per species, 154 families that were single-copy in 10 sequenced green algal genomes and the outgroup species *Arabidopsis thaliana*, *Oryza sativa* and *Physcomitrella patens*, were extracted (see Supplementary dataset 1). For every single-copy core gene family, a multiple alignment was created using MUSCLE [14]. Alignment columns containing gaps were removed when a gap was present in >10% of the sequences. Alignment columns containing gaps were removed when a gap was present in >10% of the sequences. To reduce the chance of including misaligned amino acids, all positions in the alignment left or right from the gap were also removed until a column in the sequence alignment was found where the residues were conserved in all genes included in our analyses. This was determined as follows: for every pair of residues in the column, the BLOSUM62 value was retrieved. Next, the median value for all these values was calculated. If this median was ≥0, the column was considered as containing homologous amino acids. The different edited multiple alignments were concatenated into one super-alignment using a custom Perl script (35,431 amino acids, see Supplementary dataset 2) and used to construct a phylogenetic tree (Fig. S1) using PhyML (100 bootstrap sets, WAG model, kappa estimated, 4 substitution rate categories, gamma distribution parameter estimated, BIONJ starting tree, no topology, branch lengths and rate parameter optimization) [15].

**Analysis of SOC in *Ostreococcus sp*. RCC809.** From the current RCC809 genome assembly, the most likely SOC scaffold would be chromosome_18. However, it contains a large colinear region with chromosome 10 of *Ostreococcus tauri*, a feature that does not fit with the description of SOCs in the other *Ostreococcus* genomes. The definitive nature of the RCC809 SOC therefore remains speculative.

**References**

# Keller MD, Selvin RC, Claus W, Guillard RRL: Media for the culture of oceanic ultraphytoplantkon*.* J. Phycol 1987, 23:633-638.

1. Winnepenninckx B, Backeljau T, De Wachter R: [Extraction of high molecular weight DNA from molluscs.](http://www.ncbi.nlm.nih.gov/pubmed/8122306) Trends Genet 1993, 9:407
2. Blanc G, Duncan G, Agarkova I, Borodovsky M, Gurmon J, Kuo A et al: The *Chlorella variabilis* NC64A Genome Reveals Adaptation to Photosymbiosis, Coevolution with Viruses, and Cryptic Sex Plant Cell 2010, 22:2943-2955
3. Smit AFA, Hubley R, Green P Repeat Masker Open. 3.0. 1996-2010

# [Jurka, J](http://www.ncbi.nlm.nih.gov/pubmed?term=%22Jurka%20J%22%5BAuthor%5D) [Kapitonov VV](http://www.ncbi.nlm.nih.gov/pubmed?term=%22Kapitonov%20VV%22%5BAuthor%5D), [Pavlicek A](http://www.ncbi.nlm.nih.gov/pubmed?term=%22Pavlicek%20A%22%5BAuthor%5D), [Klonowski P](http://www.ncbi.nlm.nih.gov/pubmed?term=%22Klonowski%20P%22%5BAuthor%5D), [Kohany O](http://www.ncbi.nlm.nih.gov/pubmed?term=%22Kohany%20O%22%5BAuthor%5D), [Walichiewicz J](http://www.ncbi.nlm.nih.gov/pubmed?term=%22Walichiewicz%20J%22%5BAuthor%5D): Repbase Update, a database of eukaryotic repetitive elements. [Cytogenet Genome Res](http://www.ncbi.nlm.nih.gov/pubmed?term=16093699) 2005, 110:462-427

# [Ellinghaus D](http://www.ncbi.nlm.nih.gov/pubmed?term=%22Ellinghaus%20D%22%5BAuthor%5D), [Kurtz S](http://www.ncbi.nlm.nih.gov/pubmed?term=%22Kurtz%20S%22%5BAuthor%5D), [Willhoeft U](http://www.ncbi.nlm.nih.gov/pubmed?term=%22Willhoeft%20U%22%5BAuthor%5D): LTRharvest, an efficient and flexible software for de novo detection of LTR retrotransposons. [BMC Bioinformatics](http://www.ncbi.nlm.nih.gov/pubmed?term=18194517) 2008, 14:9:18

# [Steinbiss S](http://www.ncbi.nlm.nih.gov/pubmed?term=%22Steinbiss%20S%22%5BAuthor%5D), [Willhoeft U](http://www.ncbi.nlm.nih.gov/pubmed?term=%22Willhoeft%20U%22%5BAuthor%5D), [Gremme G](http://www.ncbi.nlm.nih.gov/pubmed?term=%22Gremme%20G%22%5BAuthor%5D), [Kurtz S](http://www.ncbi.nlm.nih.gov/pubmed?term=%22Kurtz%20S%22%5BAuthor%5D): Fine-grained annotation and classification of de novo predicted LTR retrotransposons. [Nucleic Acids Res](http://www.ncbi.nlm.nih.gov/pubmed?term=19786494) 2009, 37:7002-7013

# [Llorens C](http://www.ncbi.nlm.nih.gov/pubmed?term=%22Llorens%20C%22%5BAuthor%5D), [Futami R](http://www.ncbi.nlm.nih.gov/pubmed?term=%22Futami%20R%22%5BAuthor%5D), [Covelli L](http://www.ncbi.nlm.nih.gov/pubmed?term=%22Covelli%20L%22%5BAuthor%5D), [Domínguez-Escribá L](http://www.ncbi.nlm.nih.gov/pubmed?term=%22Dom%C3%ADnguez-Escrib%C3%A1%20L%22%5BAuthor%5D), [Viu JM](http://www.ncbi.nlm.nih.gov/pubmed?term=%22Viu%20JM%22%5BAuthor%5D), [Tamarit D](http://www.ncbi.nlm.nih.gov/pubmed?term=%22Tamarit%20D%22%5BAuthor%5D), [Aguilar-Rodríguez J](http://www.ncbi.nlm.nih.gov/pubmed?term=%22Aguilar-Rodr%C3%ADguez%20J%22%5BAuthor%5D), [Vicente-Ripolles M](http://www.ncbi.nlm.nih.gov/pubmed?term=%22Vicente-Ripolles%20M%22%5BAuthor%5D), [Fuster G](http://www.ncbi.nlm.nih.gov/pubmed?term=%22Fuster%20G%22%5BAuthor%5D), [Bernet GP](http://www.ncbi.nlm.nih.gov/pubmed?term=%22Bernet%20GP%22%5BAuthor%5D), [Maumus F](http://www.ncbi.nlm.nih.gov/pubmed?term=%22Maumus%20F%22%5BAuthor%5D), [Munoz-Pomer A](http://www.ncbi.nlm.nih.gov/pubmed?term=%22Munoz-Pomer%20A%22%5BAuthor%5D), [Sempere JM](http://www.ncbi.nlm.nih.gov/pubmed?term=%22Sempere%20JM%22%5BAuthor%5D), [Latorre A](http://www.ncbi.nlm.nih.gov/pubmed?term=%22Latorre%20A%22%5BAuthor%5D), [Moya A](http://www.ncbi.nlm.nih.gov/pubmed?term=%22Moya%20A%22%5BAuthor%5D): The Gypsy Database (GyDB) of mobile genetic elements: release 2.0. [Nucleic Acids Res](http://www.ncbi.nlm.nih.gov/pubmed?term=21036865) 2011, 39(Database issue):D70-74

# [Becher V](http://www.ncbi.nlm.nih.gov/pubmed?term=%22Becher%20V%22%5BAuthor%5D), [Deymonnaz A](http://www.ncbi.nlm.nih.gov/pubmed?term=%22Deymonnaz%20A%22%5BAuthor%5D), [Heiber P](http://www.ncbi.nlm.nih.gov/pubmed?term=%22Heiber%20P%22%5BAuthor%5D): Efficient computation of all perfect repeats in genomic sequences of up to half a gigabyte, with a case study on the human genome. [Bioinformatics](http://www.ncbi.nlm.nih.gov/pubmed?term=19451169) 2009, 25:1746-1753

# [Zdobnov EM](http://www.ncbi.nlm.nih.gov/pubmed?term=%22Zdobnov%20EM%22%5BAuthor%5D), [Apweiler R](http://www.ncbi.nlm.nih.gov/pubmed?term=%22Apweiler%20R%22%5BAuthor%5D): InterProScan--an integration platform for the signature-recognition methods in InterPro. [Bioinformatics](http://www.ncbi.nlm.nih.gov/pubmed?term=11590104) 2001, 17: 847-8

# [Lagesen K](http://www.ncbi.nlm.nih.gov/pubmed?term=%22Lagesen%20K%22%5BAuthor%5D), [Hallin P](http://www.ncbi.nlm.nih.gov/pubmed?term=%22Hallin%20P%22%5BAuthor%5D), [Rødland EA](http://www.ncbi.nlm.nih.gov/pubmed?term=%22R%C3%B8dland%20EA%22%5BAuthor%5D), [Staerfeldt HH](http://www.ncbi.nlm.nih.gov/pubmed?term=%22Staerfeldt%20HH%22%5BAuthor%5D), [Rognes T](http://www.ncbi.nlm.nih.gov/pubmed?term=%22Rognes%20T%22%5BAuthor%5D), [Ussery DW](http://www.ncbi.nlm.nih.gov/pubmed?term=%22Ussery%20DW%22%5BAuthor%5D): RNAmmer: consistent and rapid annotation of ribosomal RNA genes. [Nucleic Acids Res](http://www.ncbi.nlm.nih.gov/pubmed?term=17452365) 2007, 35: 3100-3108

# [Lowe TM](http://www.ncbi.nlm.nih.gov/pubmed?term=%22Lowe%20TM%22%5BAuthor%5D), [Eddy SR](http://www.ncbi.nlm.nih.gov/pubmed?term=%22Eddy%20SR%22%5BAuthor%5D): tRNAscan-SE: a program for improved detection of transfer RNA genes in genomic sequence. [Nucleic Acids Res](http://www.ncbi.nlm.nih.gov/pubmed?term=9023104) 1997, 25:955-64

# [Nawrocki EP](http://www.ncbi.nlm.nih.gov/pubmed?term=%22Nawrocki%20EP%22%5BAuthor%5D), [Kolbe DL](http://www.ncbi.nlm.nih.gov/pubmed?term=%22Kolbe%20DL%22%5BAuthor%5D), [Eddy SR](http://www.ncbi.nlm.nih.gov/pubmed?term=%22Eddy%20SR%22%5BAuthor%5D): Infernal 1.0: inference of RNA alignments. [Bioinformatics](http://www.ncbi.nlm.nih.gov/pubmed?term=19307242) 2009, 25:1335-1337

# Edgar RC: MUSCLE: multiple sequence alignment with high accuracy and high throughput. Nucleic Acids Res 2004, 32:1792-1797

1. Guindon S, Dufayard JF, Lefort V, Anisimova M, Hordijk W, Gascuel O: New algorithms and methods to estimate maximum-likelihood phylogenies: assessing the performance of PhyML 3.0. Syst Biol 2010, 59:307-321

**Fig. S1.** Maximum likelihood tree depicting the phylogenetic position of *Bathycoccus* RCC1105.


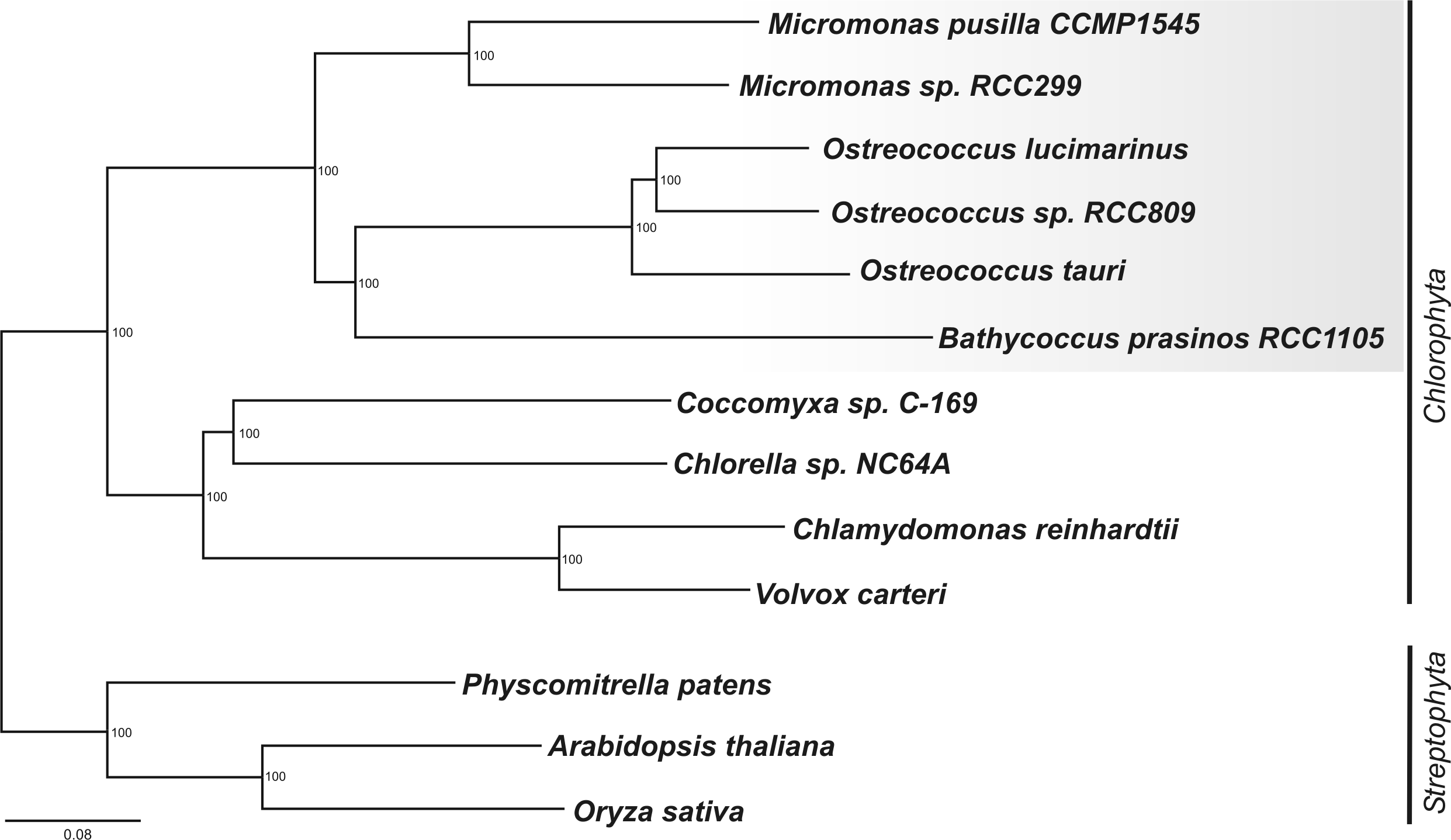


A total of 154 single-copy genes conserved in 13 species including plants were concatenated and aligned over 35,431 amino acid positions to construct the phylogeny tree using MUSCLE and PhyML (see details in Supplementary Methods). Species in the order Mamiellales are indicated by the grey box.

**Fig. S2.** Growth curve of *Bathycoccus sp.* Strain RCC1105 for the extraction of RNA to prepare cDNA libraries and sequence ESTs.

Arrow: sampling stage for the RNA extraction. Genomic DNA was prepared from a similar culture and extraction was also done at the cell concentration around 4.10^7^ cells/ml.

**Fig. S3.** Size distribution of the contigs obtained after assembly of the *Bathycoccus* genome sequencing

After assembling, sequence data were grouped in 126 contigs ranging from 3 kb to 1353 kb. The 102 smallest of these contigs were bacterial contaminations according to the blast results whereas the 24 remaining bigger contigs were part of the *Bathycoccus* genome (22 nuclear, 1 chloroplastic and mitochondrial contigs). Among the 22 nuclear contigs, six could be joined two by two giving 19 scaffolds corresponding to 19 chromosomes observed by pulse field electrophoresis.

**Fig. S4.** *Bathycoccus prasinos* RCC1105 whole-genome dotplots with *Ostreococcus lucimarinus* (upper panel) and *Micromomas sp.* RCC299 (lower panel).


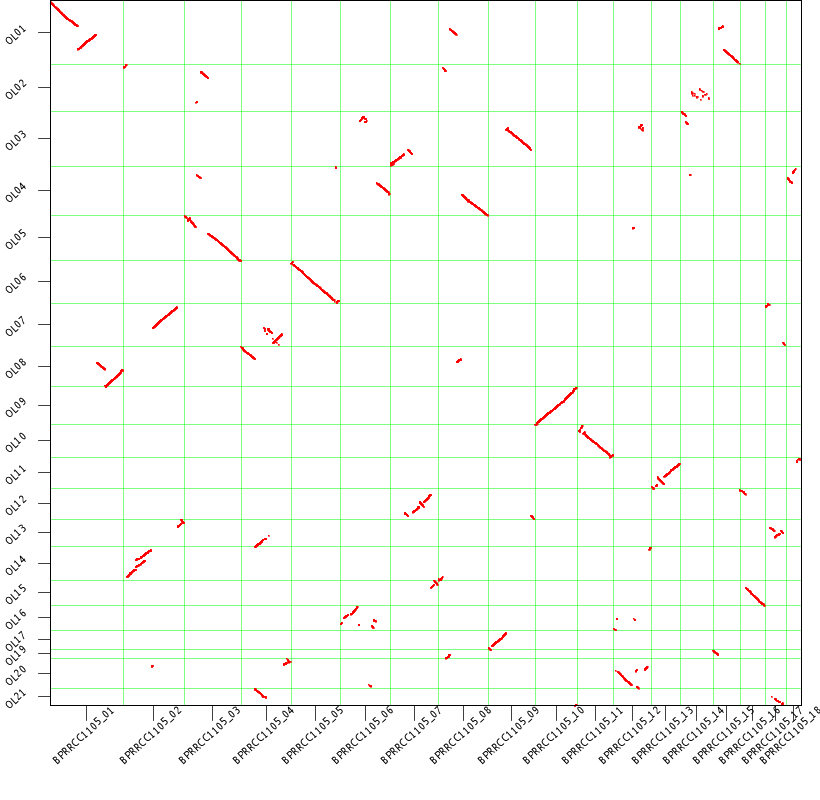

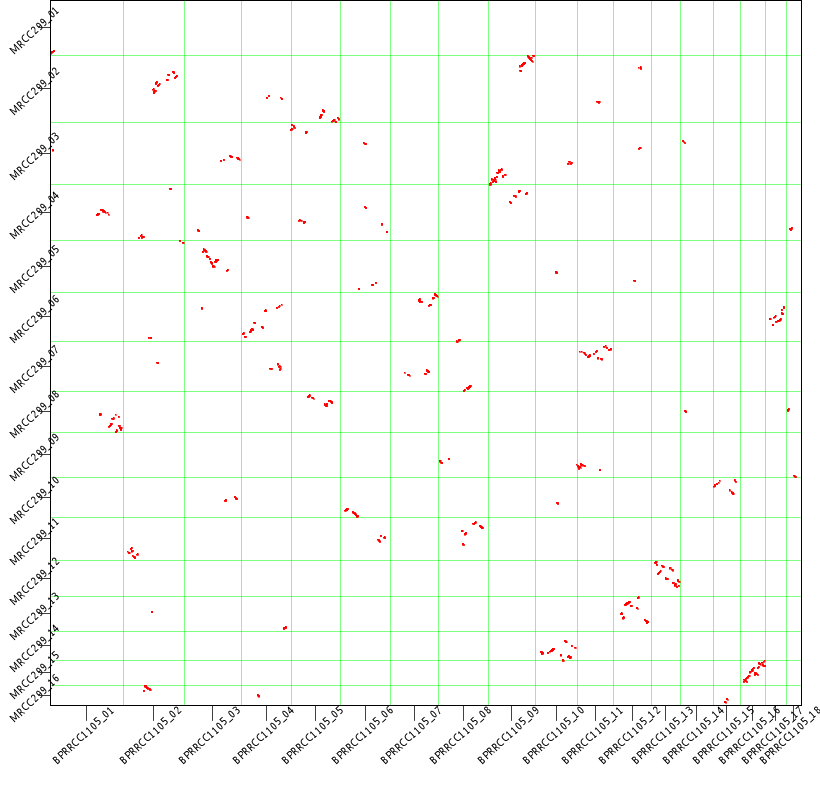


For each species all genes are depicted per chromosome (green lines) and colinear regions containing five or more genes are displayed as red dots or diagonal lines.

**Figure S5.** Pan and core genome plots for three land plant and ten sequenced green algae.


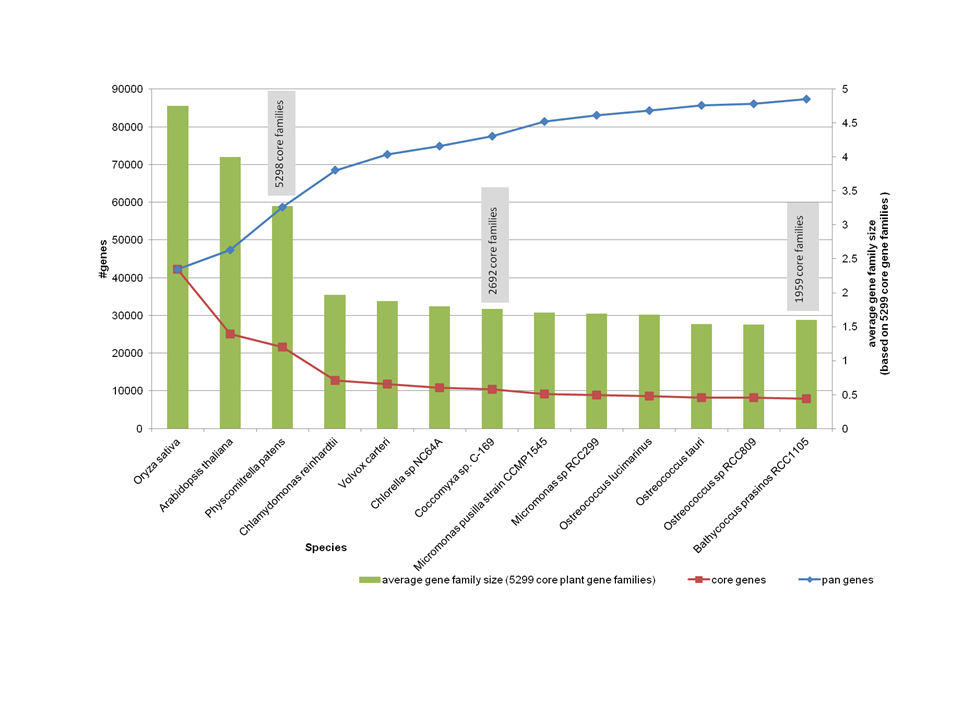

Starting from all rice proteins (reference species left), sequence similarity searches (BLASTP E-value <1e-05) were performed to determine homologous genes and core gene families in other species. Reversely, pan genes refer to new genes for which no homologs exist in the species that were already compared (from left to right). The green bars indicate the average gene family size based on a set of 5299 core gene families delineated using Tribe-MCL. Protein-coding genes for the different species were retrieved from pico-PLAZA (http://bioinformatics.psb.ugent.be/pico-plaza/).

**Figure S6.** Gene family analysis. For each clade all genes were collected, the corresponding gene families were retrieved and singletons were removed.


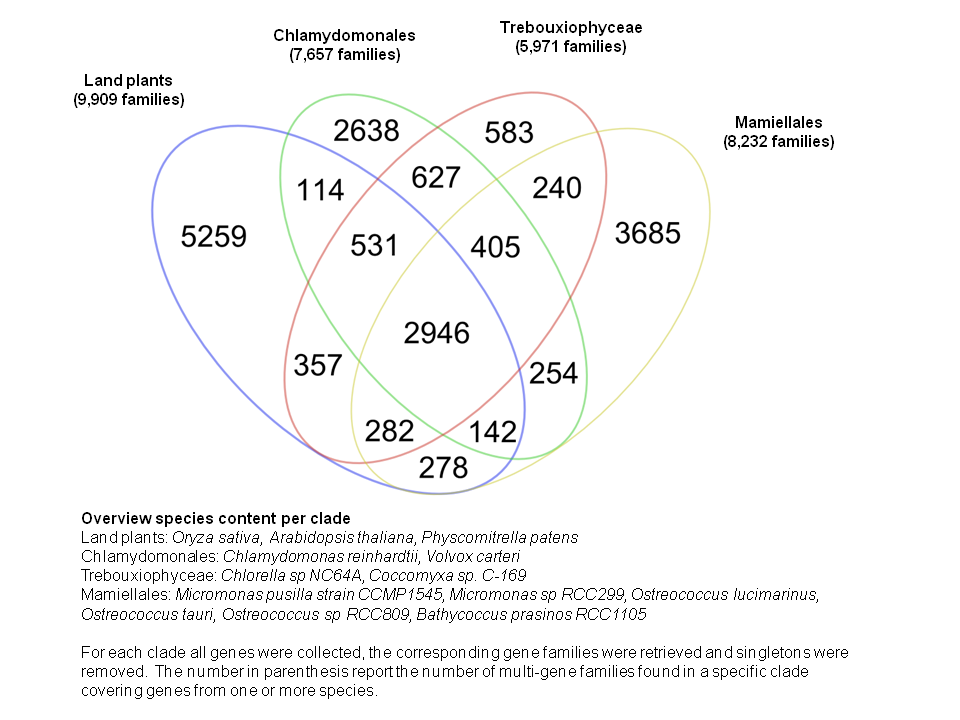


The numbers in parenthesis report the number of multi-gene families found in a specific clade covering genes from one or more species (i.e. families not necessarily exist in all species of a clade). Protein-coding genes and gene families were retrieved using the pico-PLAZA Gene Family Finder (<http://bioinformatics.psb.ugent.be/pico-plaza/>).

**Figure S7.** GC content of outlier chromosomes in Mamiellales genomes.


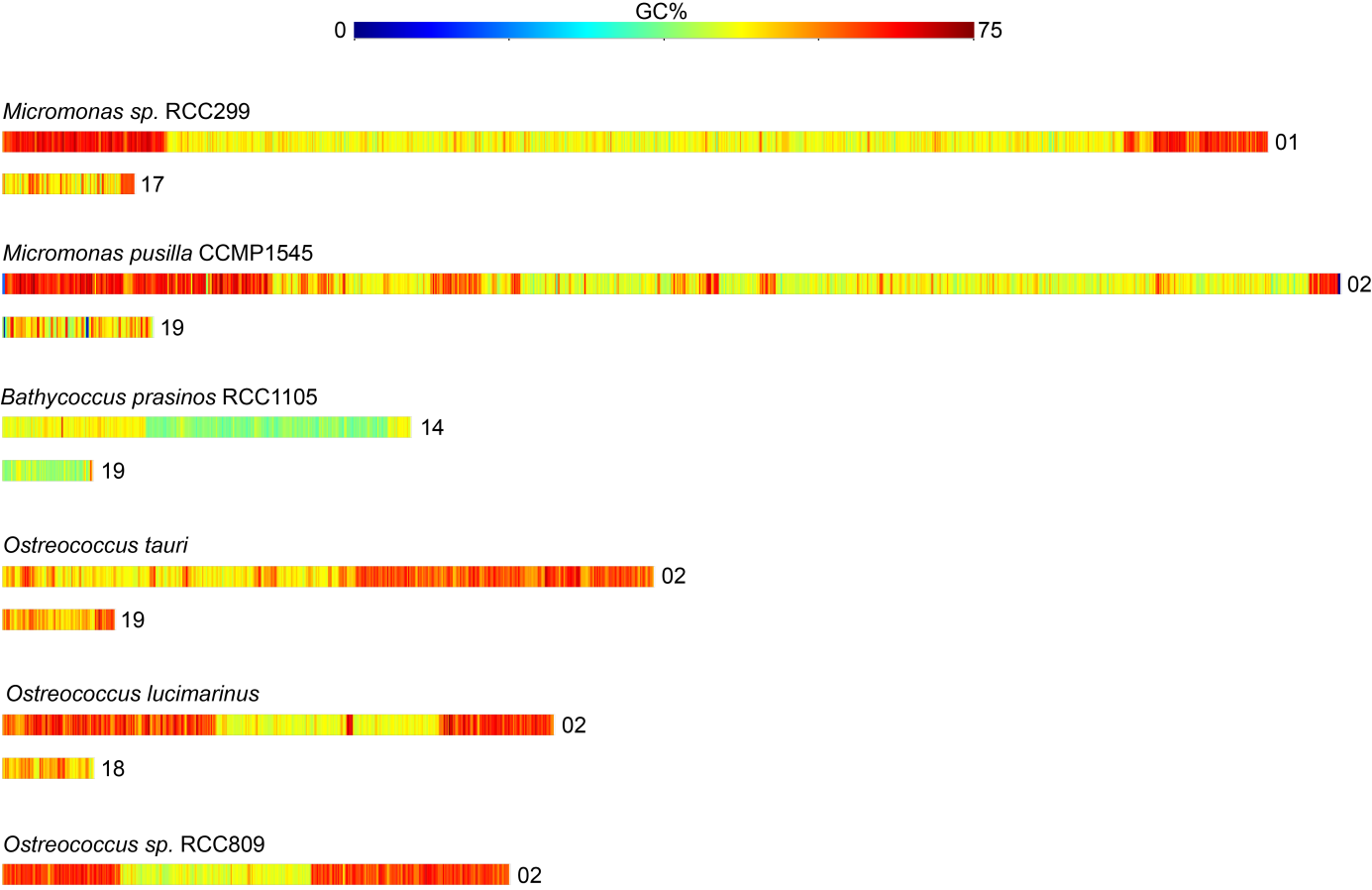


The GC content is plotted using a window size of 2kb. The numbers at the end of each bar indicate the chromosome number. We define the BOC1 region in *Bathycoccus* as that spanning nucleotide positions 236,365 to 624,661.

**Figure S8.** Function and expression analysis for BOC1 genes.


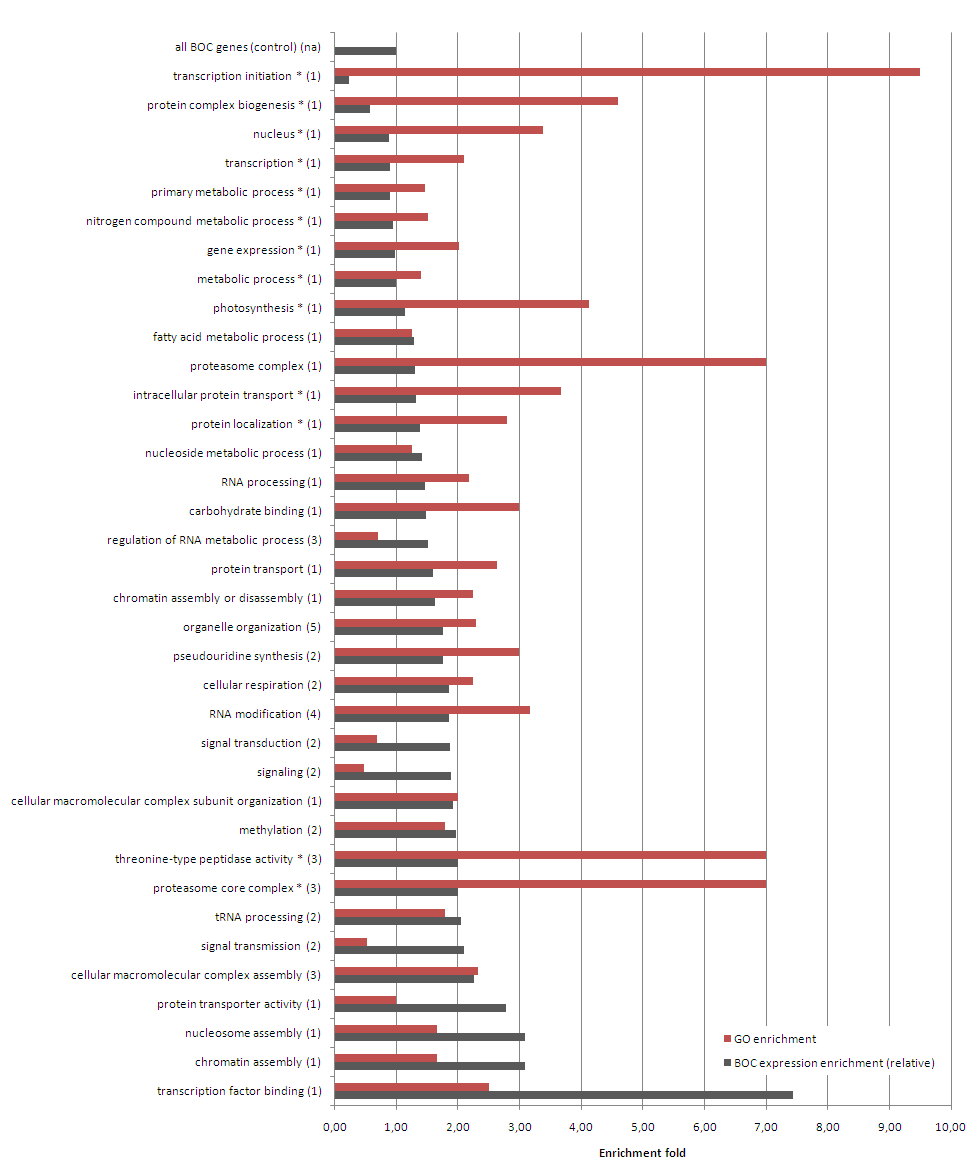


The red bars show Gene Ontology enrichment while the black bars indicate increased expression per functional category. Asterisks indicate GO categories with significant enrichment in BOC1 whereas the number of genes per functional category is reported in parenthesis.

**Figure S9.** Gene expression of BOC1, Rest and SOC genes in Mamiellales and non-Mamiellales green algae.

A.


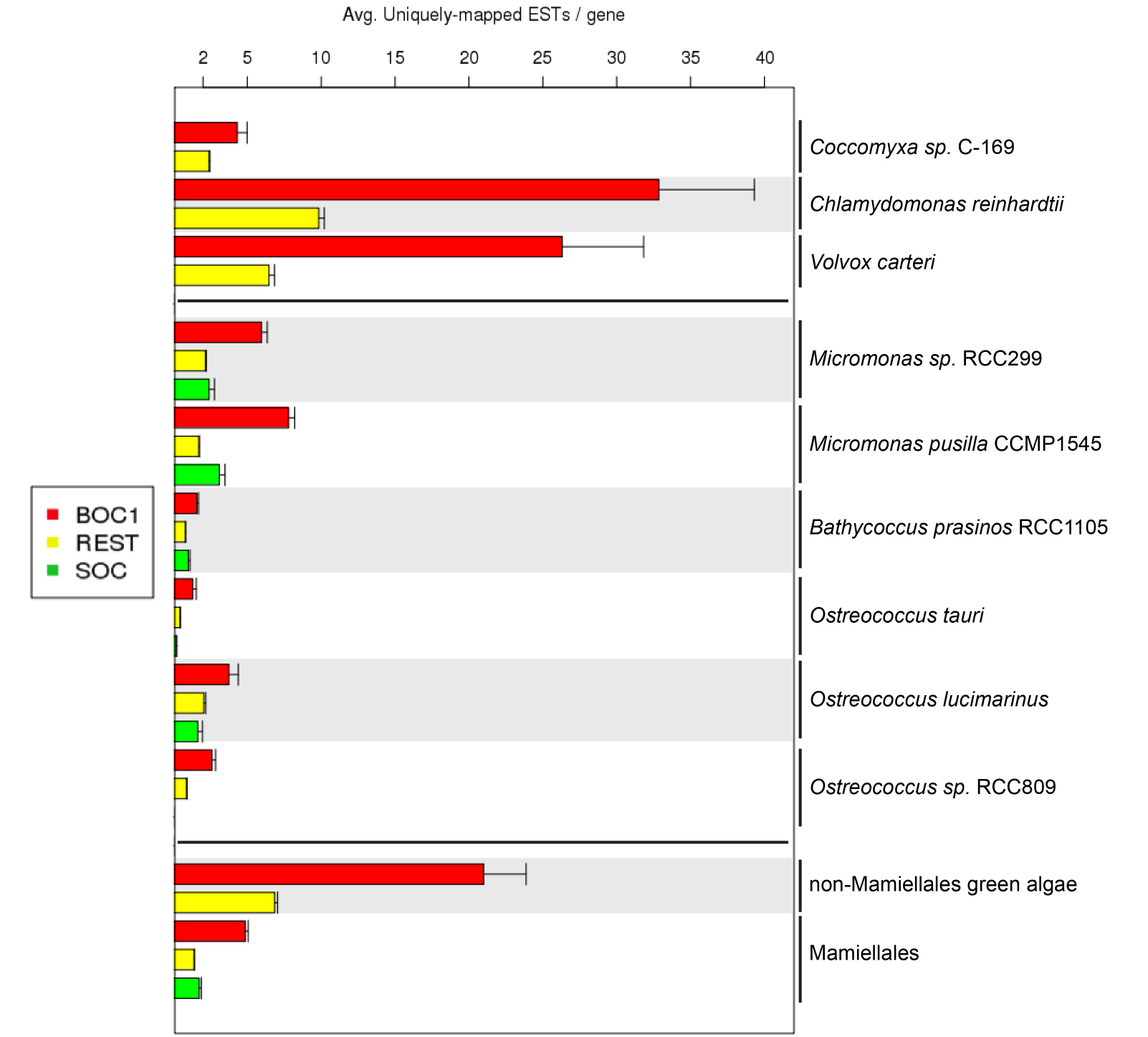


B.


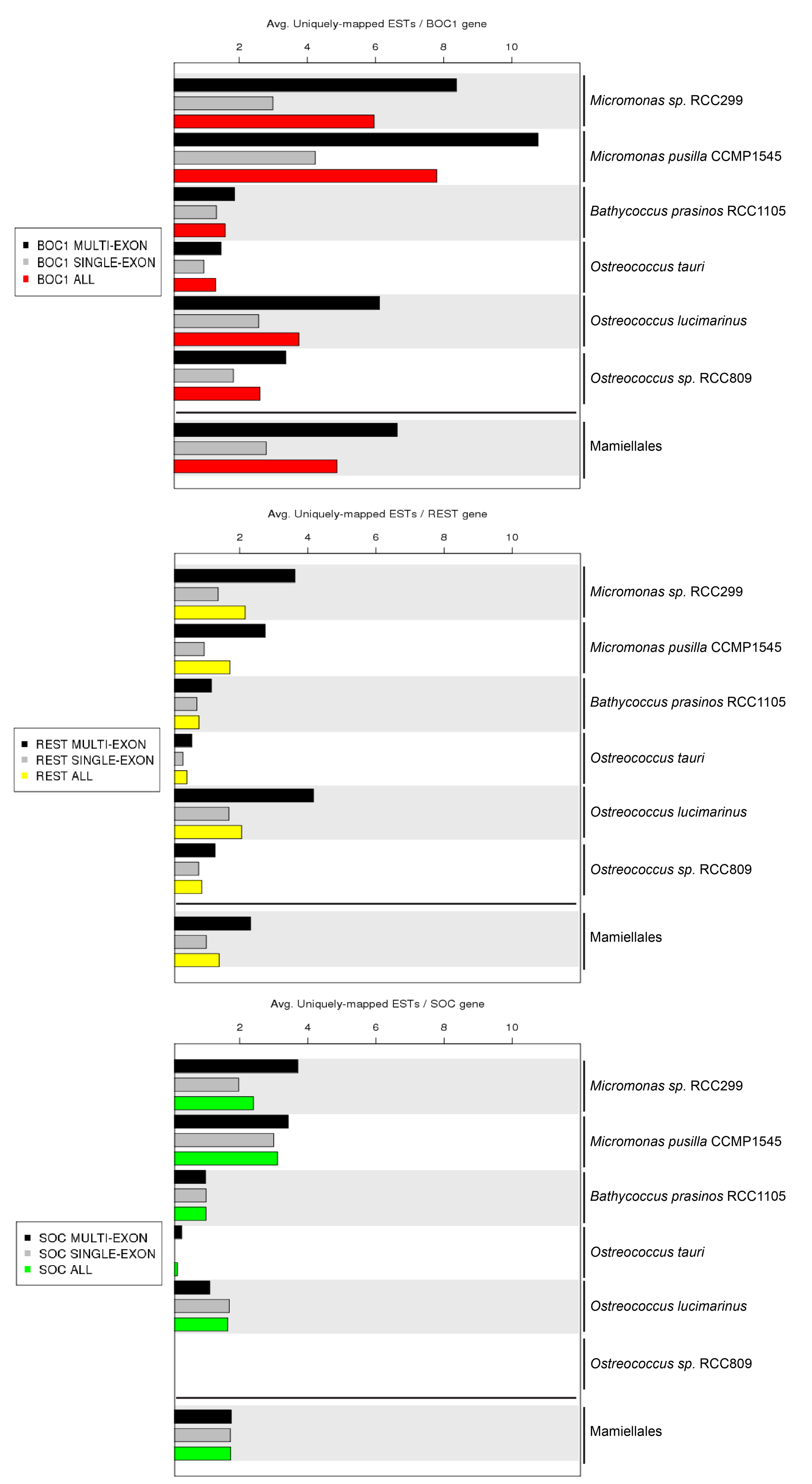


(A) For non-Mamiellales, a virtual BOC1 region was created by grouping all the best BLASTP hits for each *Bathycoccus prasinos* RCC1105 BOC1 gene. REST refers to genes not belonging to BOC1 and SOC, respectively. This procedure could not be repeated for SOC, as this region contains too many species-specific genes. Error bars indicate SE. (B) Gene expression quantification for BOC1, Rest and SOC gene sets with and without introns.

**Figure S10.** Intron length distribution in Mamiellales and non-Mamiellales green algae.


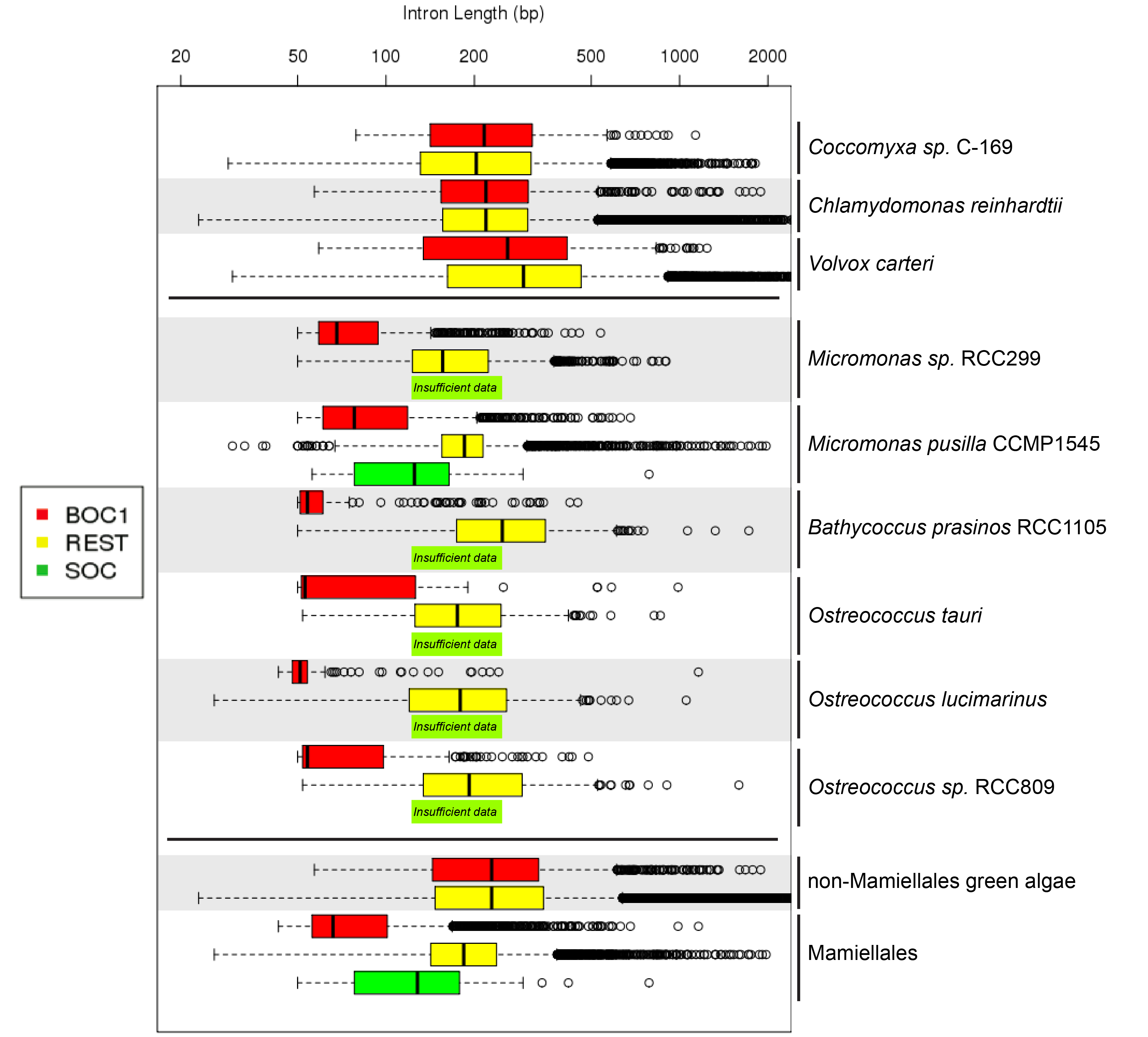


For each organism, the lengths of BOC1, REST and SOC EST-confirmed introns are shown. For the BOC1 definition and SOC absence in non-Mamiellales, see Fig. S3. ‘Insufficient data’ indicates either an absence of EST-confirmed introns or too few data points (less than 11) to construct a boxplot. The data clearly shows that SOC genes carry little (EST-confirmed) introns. For the sake of visibility, intron length outliers above 2000bp are not displayed.

**Figure S11.** Maximum likelihood phylogenetic tree for an expanded gene family including sialyltransferases (HOM000519 in the pico-PLAZA platform).

Gene models are displayed using blue and green boxes, which indicate coding and UTR exons, respectively. Species prefixes indicate ath - *Arabidopsis thaliana*, osa - *Oryza sativa*, ppa - P*hyscomitrella patens* and bprrcc1105 - *Bathycoccus prasinos RCC1105*. Symbols “e” (blue) and “u” (green) refer to coding exons and UTR, respectively.
**Figure S12.** Genome-wide mapping *Bathycoccus* for the Ankyrin repeat-containing domain genes (IPR020683).

**
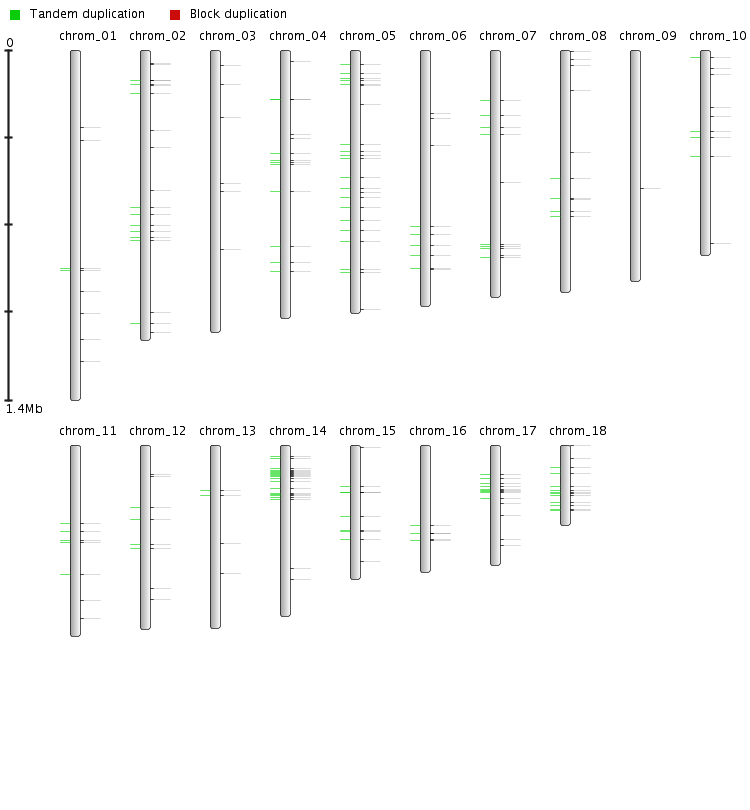
**

Location of the genes are marked by grey arrays and those which are tandemly duplicated are also marked by a green bar. There is no block duplication for these genes.

**Table S1.** General annotation statistics for *Bathycoccus prasinos* RCC1105.

|  | **Information** | | | | |
| --- | --- | --- | --- | --- | --- |
| **Genome** | 22 contigs for 19 chromosomes, 1 chloroplast, 1 mitochondrion  Genome length: 15,122,588 nt  N50*: 8  L50*: 937,610 nt  Gaps (N>20): 22  Total gap length: 36,954 nt | | | | |
| **Genes** | **Gene Type** | **Total genes** | **Nuclear genes** | **Mitochondrion genes** | **Chloroplast genes** |
|  | Coding | 7,919 | 7,826 | 41 | 52 |
|  | tRNA | 57 | 17 | 26 | 14 |
|  | rRNA | 10 | 4 | 4 | 2 |
|  | Total | 7,986 | 7,847 | 71 | 68 |
|  | **Gene property** | **Number of Genes**  **(% of total Genes)** | | | |
|  | Multi-exon | 1174 (14.70) | | | |
|  | EST-support | 3692 (46.23) | | | |
|  | Homology-support ^2^ | 6789 (85.01) | | | |
|  | InterPro domains | 6160 (77.13) | | | |
|  | GO-labels | 3597 (45.04) | | | |

* L50, length of the scaffold that separates the top half (N50) of the assembled genome from the remainder of the smaller scaffolds, if the sequences are ordered by size. N50 is the number of scaffolds that represent the top half of the assembled genomes, if the sequences are ordered by size.

**Table S2.** Annotation of the BOC1 region in different Mamiellales species.

| **Species** | **chromosome** | **BOC1 start** | **BOC1 end** | **Length (bp)** | **GC%** |
| --- | --- | --- | --- | --- | --- |
| *Bathycoccus sp. prasinos* | 14 | 236365 | 624661 | 388296 | 39 |
| *Micromonas sp. RCC299* | 1 | 263000 | 1817000 | 1554001 | 47 |
| *Micromonas sp*. CCMP1545 | 2 | 438300 | 2112000 | 1673701 | 48 |
| *Ostreococcus lucimarinus* | 2 | 345000 | 709200 | 364201 | 47 |
| *Ostreococcus sp.* RCC809 | 2 | 180000 | 500000 | 320001 | 46 |
| *Ostreococcus tauri* | 2 | 1 | 575000 | 575000 | 50 |

**Table S3.** *Bathycoccus* BOC1 Mamiellales core genes and their functional description.

| **Locus_id** | **Functional description** |
| --- | --- |
| Bathy14g01300 | beta-adaptin-like protein C |
| Bathy14g01380 | TFIID component TAF4 |
| Bathy14g01390 | Phosphotyrosyl phosphatase activator, PTPA |
| Bathy14g01470 | U3 small nucleolar RNA-associated protein 18 |
| Bathy14g01520 | arginyl-tRNA synthetase |
| Bathy14g01530 | glycosyltransferase family 28 protein, putative Monogalactosyldiacylglycerol (MGDG) synthase |
| Bathy14g01650 | Mg-protoporyphyrin IX chelatase |
| Bathy14g01670 | Phosphatidic acid Phosphatase-related protein |
| Bathy14g01700 | glycosyltransferase family 4 protein, putative alpha-1,3-mannosyltransferase ALG2 |
| Bathy14g01860 | Caf1 CCR4-associated (transcription) factor 1 |
| Bathy14g02130 | ribosome biogenesis protein RLP24 |
| Bathy14g02140 | coatomer protein gamma-subunit |
| Bathy14g02190 | CycK-related cyclin family protein |
| Bathy14g02270 | eukaryotic translation initiation factor 4E |
| Bathy14g02340 | histidinol-phosphate aminotransferase, chloroplast precursor |
| Bathy14g02350 | transcription factor IIa large subunit 3 |
| Bathy14g02360 | MAK16-like protein |
| Bathy14g02380 | Isoleucine-tRNA synthetase, probable |
| Bathy14g02640 | ATP synthase beta chain, mitochondrial precursor |
| Bathy14g02730 | V-type proton ATPase subunit d 1 |
| Bathy14g02790 | 60S ribosomal protein L36 |
| Bathy14g02810 | U3 small nucleolar RNA-associated protein 6 |
| Bathy14g03000 | Ribosome biogenesis protein BOP1 |
| Bathy14g03050 | UphC Sugar phosphate permease, putative regulatory protein |
| Bathy14g03060 | 1-deoxy-D-xylulose-5-phosphate (DXP) synthase, plastid precursor |
| Bathy14g03100 | Tim circadian rhythm control protein Timeless homolog |
| Bathy14g03180 | Conserved oligomeric Golgi complex component 4 |
| Bathy14g03200 | eukaryotic translation initiation factor 6 |
| Bathy14g03330 | RNA Polymerase subunit 2 |

**Table S4.** Significant clustering of expressed genes and multi-exon genes.

| **Organism** | **Category** | **threshold** | **Significant Cluster Region (nt)** | **P-value** |
| --- | --- | --- | --- | --- |
| *B. prasinos* RCC1105 | Expressed | #ESTs > 0 | chrom 14: 215796 - 366969 | 1.65915e-09 |
|  |  |  | chrom 14: 469215 - 621558 | 7.19417e-09 |
|  | Intron Content | #introns > 0 | chrom 14: 236365 - 378755 | 1.67806e-14 |
|  |  |  | chrom 14: 458273 - 605497 | 1.67806e-14 |
|  |  |  | chrom 14: 368396 - 501102 | 1.02555e-13 |
|  |  | #introns > 2 | chrom 14: 475097 - 621558 | 8.02247e-27 |
|  |  |  | chrom 14: 305215 - 433073 | 2.52144e-21 |
| *O. tauri* | Expressed | #ESTs > 0 | chrom 02: 475670 - 545753 | 1.44266e-09 |
|  | Intron Content | #introns > 0 | chrom 02: 281590 - 374033 | 1.57754e-13 |
|  |  |  | chrom 03: 724931 - 863076 * | 2.17355e-08 |
|  |  |  | chrom 02: 157589 - 318301 | 2.42314e-08 |
|  |  | #introns > 2 | chrom 02: 290969 - 384778 | 4.38209e-16 |
| *O. lucimarinus* | Expressed | #ESTs > 0 | chrom 02: 583161 - 683580 | 1.0409e-09 |
|  |  | #ESTs > 2 | chrom 14: 173374 - 297922 * | 1.27158e-13 |
|  | Intron Content | #introns > 0 | chrom 02: 600266 - 699849 | 1.4305e-10 |
| *O. sp.* RCC809 | Expressed | #ESTs > 0 | chrom 02: 317288 - 443407 | 4.83765e-14 |
|  | Intron Content | #introns > 0 | chrom 02: 248544 - 382410 | 6.92721e-15 |
|  |  |  | chrom 02: 406912 - 486843 | 2.5338e-13 |
|  |  |  | chrom 06: 991068 - 1046898 * | 2.72831e-08 |
|  |  | #introns > 2 | chrom 02: 320761 - 447820 | 2.52125e-19 |
|  |  |  | chrom 02: 204204 - 344730 | 4.15792e-16 |
| *M. sp.* RCC299 | Expressed | #ESTs > 0 | chrom 01: 1476814 - 1686210 | 4.52851e-17 |
|  |  |  | chrom 01: 1616446 - 1814523 | 1.8217e-16 |
|  |  |  | chrom 01: 1257091 - 1459584 | 4.63294e-16 |
|  |  | #ESTs > 2 | chrom 01: 1050872 - 1240539 | 9.25502e-22 |
|  |  |  | chrom 01: 930272 - 1123123 | 2.5469e-21 |
|  |  |  | chrom 01: 575962 - 771604 | 7.73573e-17 |
|  |  |  | chrom 01: 275485 - 449940 | 1.9546e-14 |
|  |  |  | chrom 01: 376996 - 579366 | 1.11257e-13 |
|  | Intron Content | #introns > 2 | chrom 01: 1807062 - 2000430 | 1.50583e-08 |
| *M. pusilla* CCMP1545 | Expressed | #ESTs > 0 | chrom 02: 420522 - 694975 | 1.91421e-14 |
|  |  | #ESTs > 2 | chrom 02: 840200 - 1057184 | 6.03583e-32 |
|  |  |  | chrom 02: 1254871 - 1385130 | 1.8568e-28 |
|  |  |  | chrom 02: 1925724 - 2109312 | 2.14031e-28 |
|  |  |  | chrom 02: 1657908 - 1838060 | 3.52691e-26 |
|  |  |  | chrom 02: 1535396 - 1711843 | 2.76812e-24 |
|  |  |  | chrom 02: 1783247 - 1973415 | 1.7313e-22 |
|  |  |  | chrom 02: 1002800 - 1216232 | 3.60018e-20 |
|  | Intron Content | #introns > 0 | chrom 18: 1271 - 110276 * | 3.58297e-09 |
|  |  | #introns > 2 | chrom 02: 314423 - 492562 | 1.56957e-09 |
|  |  |  | chrom 02: 130505 - 271974 * | 6.60848e-09 |

Listed here are all Mamiellales chromosomal regions in which C-hunter found a significant clustering of genes in one of the four functional categories related to expression and intron content. Cluster regions marked with an asterisk do not overlap any of the Mamiellales BOC1 regions.

**Table S5.** Summary table with putative HGT *Bathycoccus* genes

| **Taxonomy** | **All HGT genes (cov.>0, bs.>0, incl. singletons) (1)** | **Singleton** | **HGT trees with**  **bs. >= 90%** | **HGT trees with bs. >= 90%**  **and cov. >=50%** |
| --- | --- | --- | --- | --- |
| Archaea; Euryarchaeota | 2 |  | 1 | 1 |
| Bacteria; Acidobacteria | 1 | 1 |  |  |
| Bacteria; Actinobacteria | 4 | 4 |  |  |
| Bacteria; Aquificae | 1 |  |  |  |
| Bacteria; Bacteroidetes | 1 | 1 |  |  |
| Bacteria; Bacteroidetes/Chlorobi group | 7 | 1 | 2 | 1 |
| Bacteria; Chlamydiae | 1 |  |  |  |
| Bacteria; Chlamydiae/Verrucomicrobia Group | 3 | 1 |  |  |
| Bacteria; Cyanobacteria | 5 | 1 | 3 | 1 |
| Bacteria; Deinococcus-Thermus | 1 |  | 1 |  |
| Bacteria; Firmicutes | 12 | 1 | 4 | 3 |
| Bacteria; Planctomycetes | 1 |  |  |  |
| Bacteria; Proteobacteria | 30 | 2 | 11 | 8 |
| Bacteria; Spirochaetes | 1 |  |  |  |
| Bacteria; Tenericutes | 4 |  |  |  |
| Eukaryota; Alveolata | 26 | 2 | 5 | 4 |
| Eukaryota; Amoebozoa | 21 | 6 |  | 4 |
| Eukaryota; Choanoflagellida | 6 | 3 | 1 | 1 |
| Eukaryota; Cryptophyta | 2 | 1 |  |  |
| Eukaryota; Euglenozoa | 6 | 3 | 1 | 1 |
| Eukaryota; Fungi | 14 | 6 | 3 | 2 |
| Eukaryota; Heterolobosea | 9 | 5 | 1 | 1 |
| Eukaryota; Ichthyosporea | 7 | 2 |  |  |
| Eukaryota; Fungi/Metazoa group | 149 | 48 | 36 | 30 |
| Eukaryota; Parabasalia | 6 |  |  |  |
| Eukaryota; stramenopiles | 98 | 25 | 25 | 22 |
| unclassified sequences | 7 | 7 |  |  |
| Viruses; dsDNA viruses, no RNA stage | 3 | 1 |  |  |
| multi-kingdom | 694 |  | 480 | 371 |
| Total HGT genes excl. 'multi-kingdom' | 428 | 121 | 94 | 79 |
| fraction Bacteria+Archaea | 17.29% | 9.92% | 23.40% | 17.72% |
| fraction Eukaryota | 80.37% | 83.47% | 76.60% | 82.28% |
|  |  |  |  |  |

1. Abbreviations cov. and bs. indicate protein alignment coverage and bootstrap support value, respectively. The set of 428 HGT genes is available in Additional dataset 4.

**Table SVI.** Gene family analysis focusing on specific biological functions.

| **Gene families conserved in land plants and green algae but lost in all Mamiellales: 531** | | |
| --- | --- | --- |
| zinc ion binding | HOM002111 | Zinc finger, FYVE/PHD-type ; Zinc finger, PHD-finger ; Zinc finger, PHD-type |
|  | HOM005325 | Zinc finger, C2HC5-type |
|  | HOM005345 | Fanconi anemia complex, subunit FancL, WD-repeat region |
|  | HOM000723 | Zinc finger, CCCH-type |
|  | HOM001679 | Copine ; von Willebrand factor, type A ; Zinc finger, RING-type |
|  | HOM001665 | Zinc finger, PHD-type ; Zinc finger, FYVE/PHD-type ; Acyl-CoA N-acyltransferase |
|  | HOM004873 | Zinc finger, NF-X1-type |
|  | HOM005785 | D111/G-patch ; Zinc finger, C2H2-type |
|  | HOM006302 | Zinc finger, U1-C type ; Zinc finger, U1-type ; Zinc finger, C2H2-type matrin |
| zinc ion transport | HOM000785 | Zinc/iron permease ; Zinc/iron permease, fungal/plant |
| UDP-glucosyltransferase activity | HOM001287 | UDP-glucuronosyl/UDP-glucosyltransferase ; Glycosyl transferase, family 28 |
|  | HOM001151 | Glycoside hydrolase, catalytic core ; Glycoside hydrolase, subgroup, catalytic core ; Glycoside hydrolase, family 20, catalytic core |
|  | HOM000023 | UDP-glucuronosyl/UDP-glucosyltransferase |
|  | HOM003359 | Glycosyl transferase, group 1 ; Sucrose-6F-phosphate phosphohydrolase, plant/cyanobacteria ; Sucrose phosphate synthase, plant |
|  | HOM002073 | Alpha-1,4-glucan-protein synthase, UDP-forming |
| vitamin binding | HOM003274 | Pyridoxal phosphate-dependent decarboxylase ; Pyridoxal phosphate-dependent transferase, major region, subdomain 1 ; Pyridoxal phosphate-dependent transferase, major domain |
|  | HOM004665 | Aminotransferase, class I/II ; Pyridoxal phosphate-dependent transferase, major domain ; Pyridoxal phosphate-dependent transferase, major region, subdomain 1 |
|  | HOM002986 | Pyridoxal phosphate-dependent decarboxylase ; Pyridoxal phosphate-dependent transferase, major domain ; Aromatic-L-amino-acid decarboxylase |
|  | HOM005727 | Aminotransferase, class V/Cysteine desulfurase ; Pyridoxal phosphate-dependent transferase, major region, subdomain 1 ; Pyridoxal phosphate-dependent transferase, major domain |
|  | HOM006056 | Pyridoxal phosphate-dependent enzyme, beta subunit |
|  | HOM005370 | Biotin/lipoyl attachment ; Single hybrid motif ; Acetyl-CoA biotin carboxyl carrier |
|  | HOM001564 | Thiamine pyrophosphate enzyme, N-terminal TPP-binding domain ; Thiamine pyrophosphate enzyme, central domain ; Pyruvate decarboxylase/indolepyruvate decarboxylase |
|  | HOM009869 | Prolyl 4-hydroxylase, alpha subunit |
| sucrose metabolic process | HOM000502 | Glycosyl hydrolases family 32, N-terminal ; Glycoside hydrolase, family 32 ; Concanavalin A-like lectin/glucanase |
|  | HOM001322 | Carbohydrate/purine kinase ; Carbohydrate/puine kinase, PfkB, conserved site ; Ribokinase |
|  | HOM003029 | UTP--glucose-1-phosphate uridylyltransferase ; UTP--glucose-1-phosphate uridylyltransferase, subgroup |
|  | HOM003359 | Glycosyl transferase, group 1 ; Sucrose-6F-phosphate phosphohydrolase, plant/cyanobacteria ; Sucrose phosphate synthase, plant |
| fatty acid biosynthetic process | HOM000170 | FAE1/Type III polyketide synthase-like protein ; Thiolase-like ; Thiolase-like, subgroup |
|  | HOM001285 | Caleosin related |
|  | HOM003806 | ATP-grasp fold, subdomain 2 ; Succinyl-CoA synthetase-like ; ATP-grasp fold, succinyl-CoA synthetase-type |
|  |  | **Core Mamiellales-specific gene families: 449** |
| zinc ion binding | HOM005305 | Zinc finger, CCCH-type ; Optic atrophy 3-like |
|  | HOM006128 | Endoribonuclease L-PSP ; Endoribonuclease L-PSP/chorismate mutase-like ; Zinc finger, C2H2-like |
|  | HOM006828 | WD40 repeat-like-containing domain ; WD40 repeat |
|  | HOM006933 | Ubiquitin ; Ubiquitin supergroup ; Zinc finger, ZZ-type |
|  | HOM007593 | CCT domain ; Zinc finger, B-box |
|  | HOM007707 | Zinc finger, CCCH-type |
|  | HOM007722 | Zinc finger, CCHC-type ; Replication fork protection component Swi3 |
|  | HOM007946 | Zinc finger, RING-type ; Zinc finger, C3HC4 RING-type |
|  | HOM008329 | Zinc finger, CCCH-type ; SAND-like ; Transcription factor IIS, N-terminal |
|  | HOM008415 | WW/Rsp5/WWP ; Zinc finger, CCHC-type |
| drug transport | HOM007393 | Multi antimicrobial extrusion protein MatE |
|  | HOM008179 | Multi antimicrobial extrusion protein MatE |
|  | HOM008194 | Multi antimicrobial extrusion protein MatE |

Gene families can be browsed via <http://bioinformatics.psb.ugent.be/pico-plaza/> using the “Search… Gene Family” option.
