# Supplementary material for: Gene functionalities and genome structure in Bathycoccus prasinos reflect cellular specializations at the base of the green lineage
Source: Genome Biol. 2012 Aug 24;13(8):R74. doi: 10.1186/gb-2012-13-8-r74 (PMC3491373; doi:10.1186/gb-2012-13-8-r74)

Report for Arachne assembly of Bathy_ass

```
Assembly started on:   mar sep 23 10:16:43 2008.
mar sep 23 14:44:52 2008.

Data:      /env/cns/proj/projet_TK/Assemblages/ARAC/Bathy_ass
Run:       /env/cns/proj/projet_TK/Assemblages/ARAC/Bathy_ass/run6
This file: /env/cns/proj/projet_TK/Assemblages/ARAC/Bathy_ass/run6/assembly.ps
```

1 Read Statistics

Initial Read Processing						
library	input reads	excluded by base quality	excluded by vector or bacterial host sequence	other exclusion	total excluded	total remaining
GSC-TK0AAA	223577	20	17	0	37	223540
GSC-TK0AAB	112842	110	11	380	501	112341
GSC-TK0ACA	3930	1	0	181	182	3748
GSC-TK0ACB	4259	12	0	578	590	3669
total	344608	143	28	1139	1310	343298

Non-Excluded Read Statistics							
library	number of reads	trimmed read length mean $\pm$ st. dev.	genomic coverage by trimmed read bases	genomic coverage by trimmed read bases of qual $\geq$ 20	fraction paired	fraction assembled	fraction assembled along with partner
GSC-TK0AAA	223540	724 $\pm$ 63	5.39X	4.76X	98.6%	95.3%	93.0%
GSC-TK0AAB	112341	798 $\pm$ 123	2.99X	2.53X	97.4%	90.2%	84.1%
GSC-TK0ACA	3748	817 $\pm$ 127	0.10X	0.08X	95.8%	80.0%	75.9%
GSC-TK0ACB	3669	806 $\pm$ 154	0.10X	0.08X	95.6%	87.1%	81.3%
total	343298	750 $\pm$ 96	8.58X	7.45X	98.1%	93.4%	89.7%

Genomic coverage was computed relative to a genome size of 30000000, as specified in the file genome.size.

2 Contig Statistics

- There are 276 contigs, of lengths: 1122692, 1058060, 1037991, 989707, 937610, 813541, 598993, 594018, 544083, 515351, 511334, 502832, 467783, 435641, 417825, ..., 1018, 936, 837, 816, 809.
- Total contig length: 16242662 bases.
- Average contig length:
  - mean = 58850 bases;
  - length-weighted mean = 581958 bases;
  - length-weighted median (N50) = 515351 bases.

3 Supercontig Statistics

- There are 130 supercontigs, of lengths: 1351588, 1122692, 1090719, 1037991, 989707, 955474, 937610, 895346, 794368, 741603, 707135, 651659, 550158, 518365, 511334, ..., 3889, 3763, 3757, 3741, 3290.
- Total supercontig length: 16402962 bases.
- Average supercontig length:
  - mean = 126176 bases;
  - length-weighted mean = 775376 bases;
  - length-weighted median (N50) = 895346 bases.

4 Contig Coverage Statistics

- Coverage of genome: (total length of included sequence)/(genome size), where the genome size is taken to be 30000000 (the value given in the file genome.size). Coverage may be exaggerated by overlaps between included sequence. (Technical note. Where we include gaps, we do not include gaps reported as negative. This could introduce a slight inconsistency between the "coverage of genome" for "all contigs and all gaps" and the total supercontig length, as reported in the previous section.)

Coverage Statistics		
sequence included	contigs	coverage of genome
contigs $\geq$ 1024 kb	3	10.73%
contigs $\geq$ 512 kb	10	27.37%
contigs $\geq$ 256 kb	22	42.87%
contigs $\geq$ 128 kb	29	47.10%
contigs $\geq$ 64 kb	35	49.10%
contigs $\geq$ 32 kb	39	49.74%
contigs $\geq$ 16 kb	47	50.30%
contigs $\geq$ 8 kb	78	51.46%
contigs $\geq$ 4 kb	190	53.50%
contigs $\geq$ 2 kb	235	53.94%
contigs $\geq$ 1 kb	272	54.13%
all contigs	276	54.14%
all contigs, and gaps $\leq$ 100 b	276	54.15%
all contigs, and gaps $\leq$ 200 b	276	54.15%
all contigs, and gaps $\leq$ 400 b	276	54.16%
all contigs, and gaps $\leq$ 800 b	276	54.20%
all contigs, and gaps $\leq$ 1600 b	276	54.29%
all contigs, and gaps $\leq$ 3200 b	276	54.41%
all contigs, and gaps $\leq$ 6400 b	276	54.62%
all contigs, and gaps $\leq$ 12800 b	276	54.65%
all contigs and all gaps	276	54.70%

5 Library by Library Statistics

5.1 Center GSC - library TK0AAA

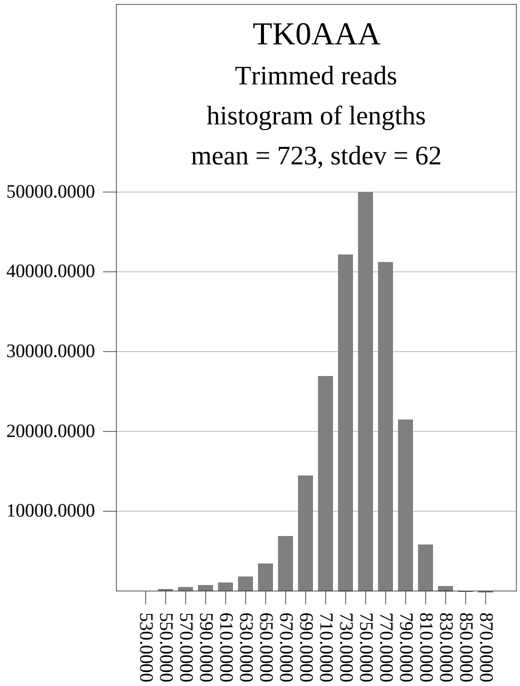

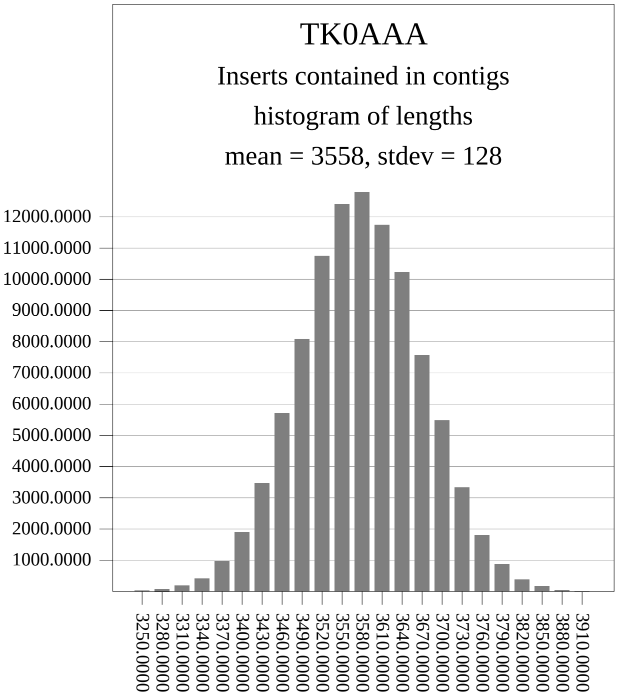

5.2 Center GSC - library TK0AAB

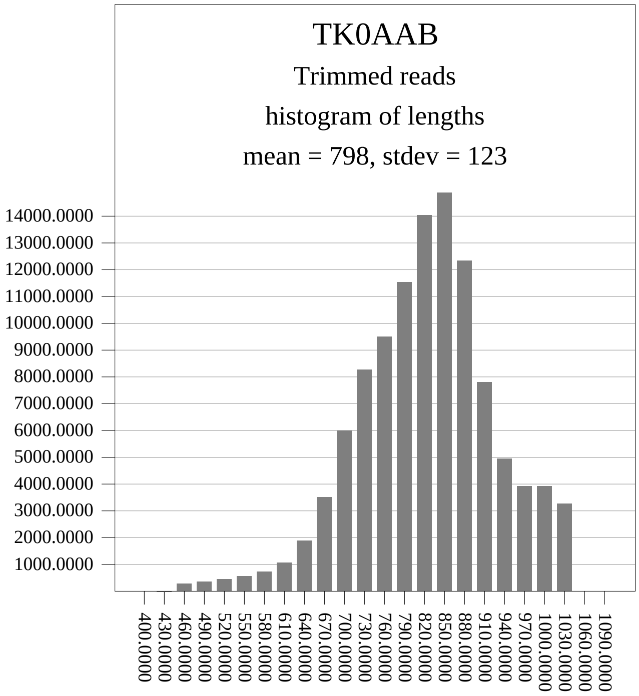

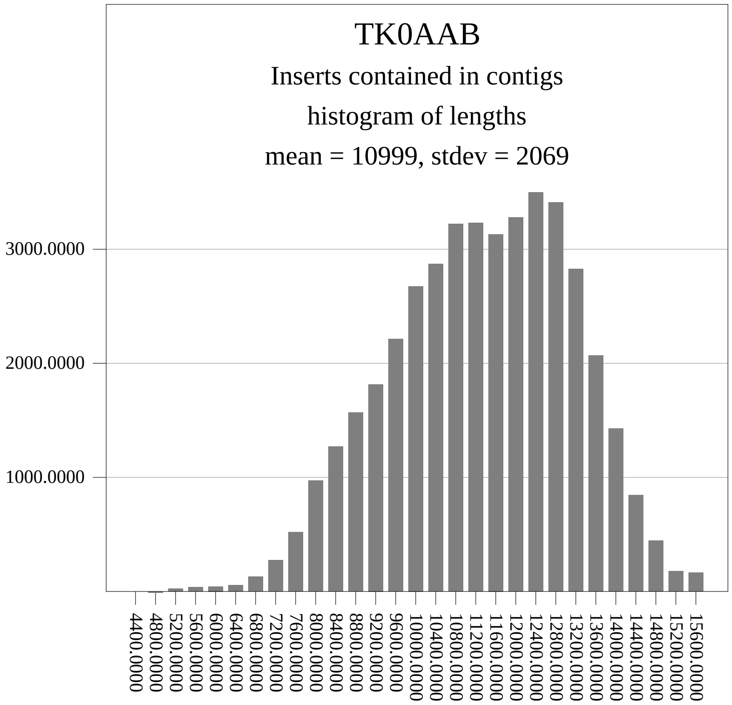

5.3 Center GSC - library TK0ACA

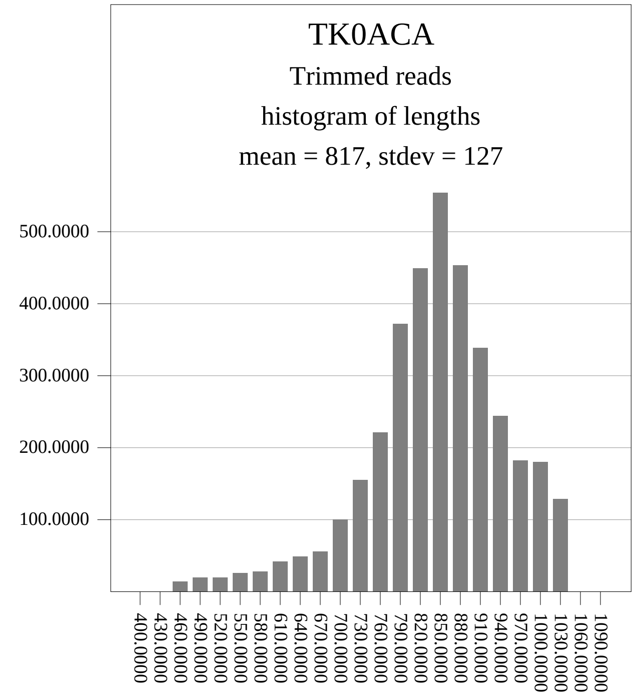

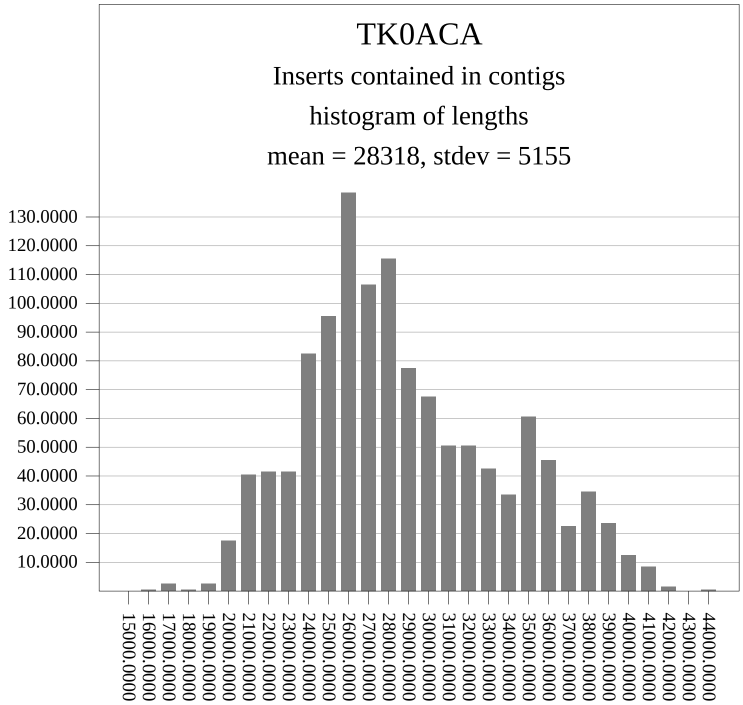

5.4 Center GSC - library TK0ACB

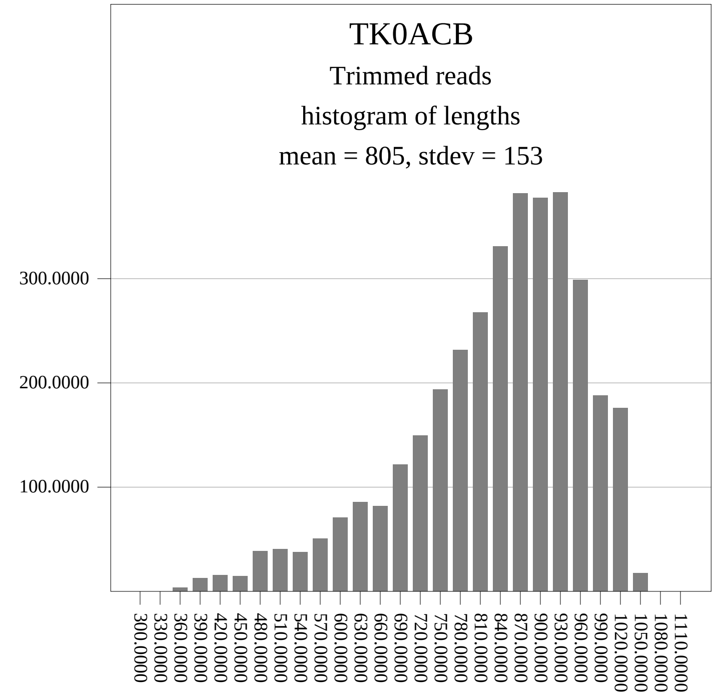

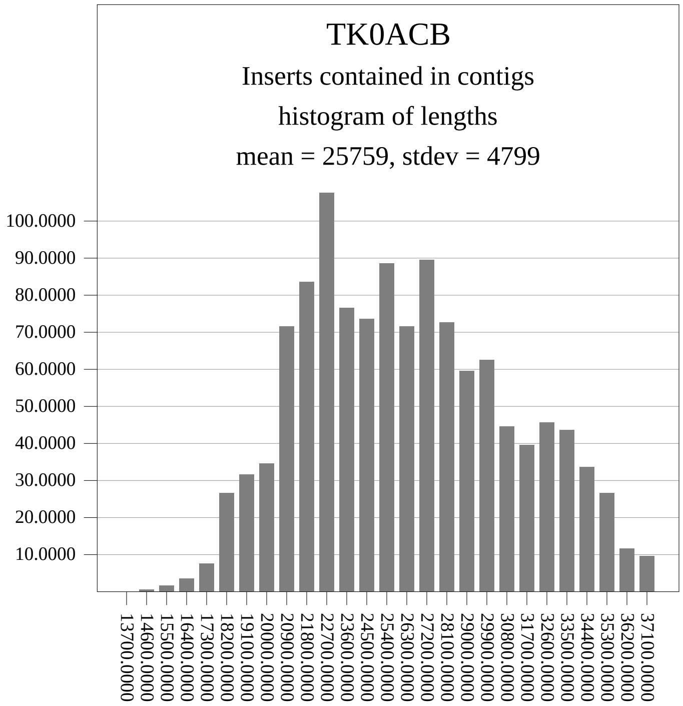

Supplement: Additional file 4 — Statistics of the genome shotgun sequencing. [file gb-2012-13-8-r74-S4.PDF]
